# Machine learning-based diagnostic and prognostic models for breast cancer: a new frontier on the clinical application of natural killer cell-related gene signatures in precision medicine

**DOI:** 10.3389/fimmu.2025.1581982

**Published:** 2025-05-27

**Authors:** Yutong Fang, Rongji Zheng, Yefeng Xiao, Qunchen Zhang, Junpeng Liu, Jundong Wu

**Affiliations:** ^1^ Department of Breast Surgery, Cancer Hospital of Shantou University Medical College, Shantou, Guangdong, China; ^2^ Department of Breast Surgery, Jiangmen Central Hospital, Jiangmen, Guangdong, China; ^3^ Department of Urology, The Second Affiliated Hospital of Shantou University, Medical College, Shantou, Guangdong, China

**Keywords:** breast cancer, natural killer cell, diagnostic model, prognostic model, machine learning

## Abstract

**Background:**

Breast cancer (BC) remains a leading cause of cancer-related mortality among women worldwide. Natural killer (NK) cells play a crucial role in the innate immune system and exhibit significant anti-tumor activity. However, the role of NK cell-related genes (NRGs) in BC diagnosis and prognosis remains underexplored. With the advent of machine learning (ML) techniques, predictive modeling based on NRGs may offer a new avenue for precision oncology.

**Methods:**

We collected transcriptomic and clinical data from The Cancer Genome Atlas (TCGA) and Gene Expression Omnibus (GEO) databases. Differentially expressed genes (DEGs) were identified, and key prognostic NRGs were selected using univariate and multivariate Cox regression analyses. We constructed ML-based diagnostic models using 12 algorithms and evaluated their performance for identifying the optimal ML diagnostic model. Additionally, a prognostic risk model was developed using LASSO-Cox regression, and its performance was validated in independent cohorts. To explore the potential mechanisms underlying the prognostic differences between high-risk and low-risk patient groups, as well as their drug treatment sensitivities, we conducted functional enrichment analysis, tumor microenvironment analysis, immunotherapy prediction, drug sensitivity analysis, and mutation analysis.

**Results:**

ULBP2, CCL5, PRDX1, IL21, NFATC2, CD2, and VAV3 were identified as key NRGs for the construction of ML models. Among the 12 ML diagnostic models, the Random Forest (RF) model demonstrated the best performance, which demonstrated robust performance in distinguishing BC from normal tissues in both training (TCGA) and validation (GEO) cohorts. In terms of the prognostic model, the risk score based on LASSO-Cox regression effectively distinguished between high-risk and low-risk patients, with patients in the high-risk group exhibiting significantly poorer overall survival (OS) compared to those in the low-risk group, and was validated in the GEO cohorts. Patients in the high-risk group displayed increased tumor proliferation, immune evasion, and reduced immune cell infiltration, correlating with poorer prognosis and lower response rates to immunotherapy. Furthermore, drug sensitivity analysis indicated that high-risk patients were more sensitive to Thapsigargin, Docetaxel, AKT inhibitor VIII, Pyrimethamine, and Epothilone B, while showing higher resistance to drugs such as I-BET-762, PHA-665752, and Belinostat.

**Conclusion:**

This study provides a comprehensive analysis of NRGs in BC and establishes reliable ML-based diagnostic and prognostic models. The findings highlight the clinical relevance of NRGs in BC progression, immune regulation, and therapy response, offering potential targets for personalized treatment strategies.

## 1 Introduction

Breast cancer (BC) is one of the leading types of cancer impacting women worldwide and is the foremost cause of cancer-related mortality among females. Recent statistics indicate that around 2.3 million new instances of BC were identified worldwide in 2022, resulting in approximately 660,000 fatalities (1). Although there are marked regional disparities in both incidence and mortality rates on a global scale, the general trend is escalating. Historically, research on BC has primarily focused on clinical manifestations and histopathological characteristics. Nevertheless, the emergence of high-throughput sequencing technologies has facilitated a paradigm shift, allowing for extensive examinations across genomic, transcriptomic, and proteomic landscapes. This advancement has unveiled intricate details concerning the molecular attributes of BC and the elaborate interplay within its tumor microenvironment (TME) (2, 3). The TME is composed of a diverse array of constituents, including immune cells, tumor-associated fibroblasts, the extracellular matrix, and the vascular system (4). These elements intricately interact, forming a sophisticated network that can either facilitate or restrain tumor progression (5). A comprehensive understanding of these components is indispensable for the development of precise and effective cancer therapies.

Natural killer (NK) cells constitute a vital component of the innate immune system and are instrumental in orchestrating anti-tumor immune responses. These cells exhibit the distinctive capability to directly eradicate tumor cells, independent of antigen-specific recognition, thus acting as a crucial cornerstone of immune surveillance (6). In addition to their direct cytotoxic action against tumor cells, NK cells assume a pivotal coordinating role within the innate immune system. By orchestrating synergistic interactions with other immune cells, they indirectly modulate the organism’s immune status and functionality (7). This coordination is essential for bolstering immune defense mechanisms and preserving immune equilibrium. Immunotherapy has achieved remarkable advancements in clinical applications, and is now extensively deployed in the treatment of various cancers. Recent advancements have led to the introduction of several innovative approaches focused on NK cells, including the development of chimeric antigen receptor NK (CAR-NK) cell therapy. This novel therapeutic modality entails the genetic modification of NK cells to express specialized chimeric antigen receptors (CARs). These receptors are tailored to detect and bind to tumor-specific antigens, significantly bolstering the NK cells’ capacity to discern and eliminate cancer cells (8). Therefore, NK cell immunotherapy offers a promising direction for the precision treatment of BC. However, the significance of NK cell-related genes (NRGs) in the diagnosis and prognosis of BC patients remains unclear, meriting further investigation.

The convergence of machine learning (ML) and medical science is catalyzing a plethora of groundbreaking innovations and transformative developments within the medical domain. ML is pivotal in clinical oncology, especially for malignancies such as BC, where it critically informs early diagnosis, strategic treatment planning, and prognostic forecasting, thereby enhancing outcomes and precision in patient tretment (9–11). Although numerous studies have employed ML algorithms to develop diagnostic or prognostic models for BC, most existing research has primarily focused on clinicopathological features and tumor-intrinsic factors, such as imaging characteristics, hormone receptor status, proliferative markers, and oncogenic signaling pathways. In contrast, relatively limited attention has been paid to the TME, particularly the role of NK cells—key components of the innate immune system. The incorporation of NRGs into ML-based models remains underexplored, overlooking the critical role of the TME in tumor progression and immune evasion. This study aims to address this gap by constructing diagnostic and prognostic models for BC utilizing ML algorithms based on NRG signatures, providing new insights into the immune landscape of BC. Our objective is to furnish innovative perspectives and robust theoretical underpinnings for the application of precision medicine in the management of BC. The significance of these models lies in their ability to elucidate the immunological underpinnings of BC while also providing strategic direction for the formulation of novel immunotherapeutic approaches. This integrative approach highlights the potential of leveraging immune system genetics to enhance the specificity and efficacy of cancer treatment modalities.

## 2 Methods

### 2.1 Data collection and candidate NRGs screening for ML models construction

**Figure 1:**
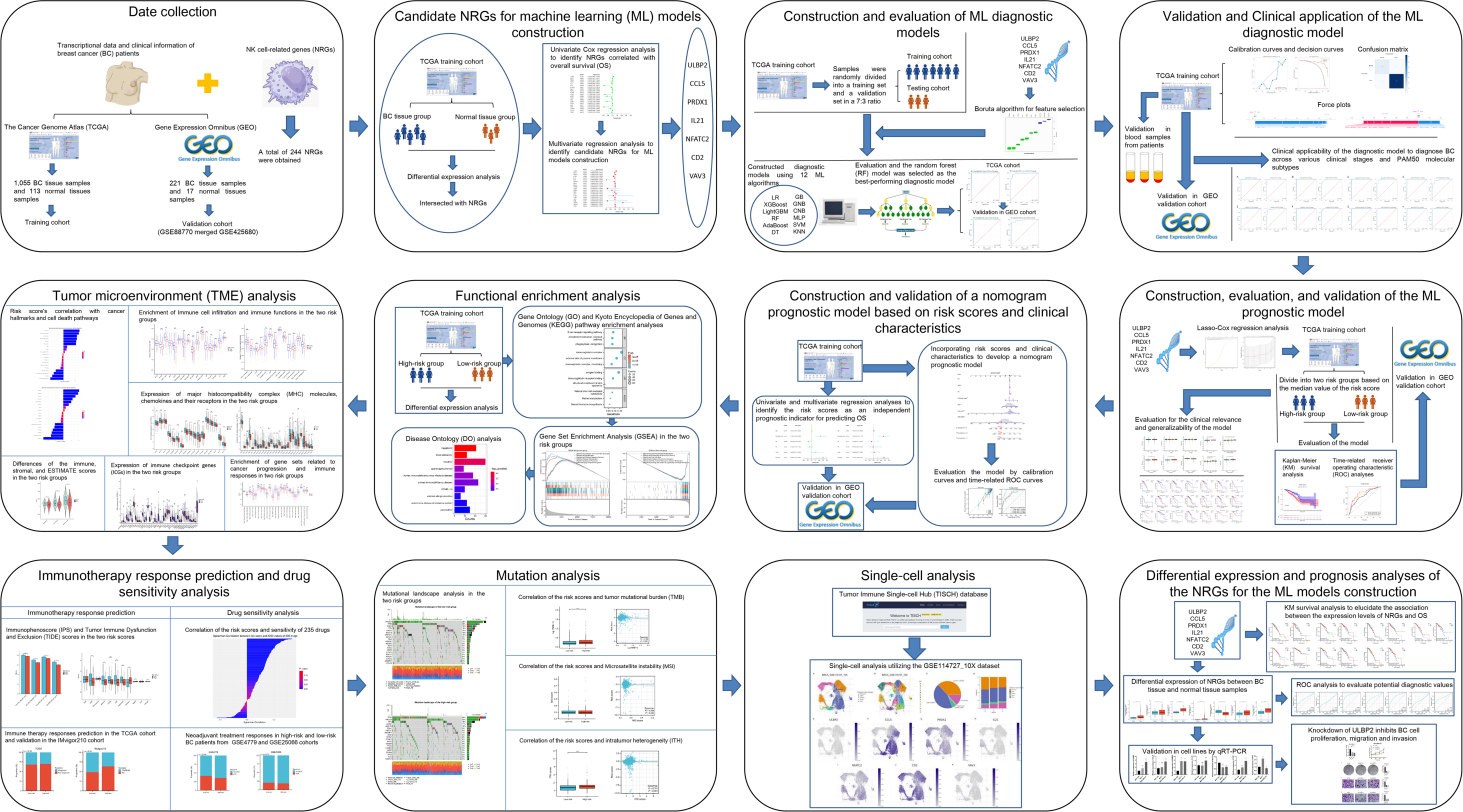
The flowchart graph depicts the methods and results in the present study.

We collected transcriptional data in FPKM format for a total of 1,113 BC tissue samples and 113 normal tissue samples from The Cancer Genome Atlas (TCGA) database (12), along with corresponding clinical information such as patient age, tumor stage, receptor status, and survival outcomes. After excluding samples with unclear prognosis information, 1,055 BC tissue samples were retained for further analysis. In addition, we merged the Gene Expression Omnibus (GEO) (13) datasets GSE42568 and GSE88770, both generated using Affymetrix Human Genome U133 Plus 2.0 Arrays, to create a combined external validation cohort for the ML-based diagnostic and prognostic models. GSE42568 comprised 17 normal and 104 BC tissues, while GSE88770 included 117 BC samples.Prior to analysis, batch normalization was applied using the “sva” R package to eliminate platform-related variability. Probe-level data were converted to gene-level expression using platform-specific annotations. Only samples with complete survival information were retained for prognostic analysis. A total of 244 NRGs (**Supplementary Table S1**) were obtained from a previously published study (14). The methods and workflow of the current study are illustrated in **Figure 1**.

To identify differentially expressed genes (DEGs) between BC and normal tissue samples, we utilized the ‘limma’ R package to conduct differential expression analysis on data from the TCGA training cohort. The criteria for DEG selection were set as |Log Fold Change| > 1 and adjusted P-value < 0.05. Subsequently, the intersections between NRGs and DEGs are identified and incorporated into a univariate Cox regression analysis aimed at selecting NRGs correlated with overall survival (OS). These identified NRGs are then subjected to multivariate regression analysis. Only those genes with a p-value less than 0.05 are deemed statistically significant and selected as candidate NRGs. These candidates will be utilized for the development of ML models that are designed to further explore and predict clinical outcomes.

### 2.2 Construction and evaluation of ML diagnostic models

After identifying the candidate NRGs, we applied the Boruta algorithm for feature selection to comprehensively assess feature importance and minimize the risk of overfitting. Following this, we developed diagnostic models using 12 ML algorithms, including logistic regression (LR), extreme gradient boosting (XGBoost), light gradient boosting machine (LightGBM), random forest (RF), adaptive boosting (AdaBoost), decision tree (DT), gradient boosting (GB), gaussian naive bayes (GNB), complement naive Bayes (CNB), multi-layer perceptron neural networks (MLP), support vector machine (SVM), and k-nearest neighbors (KNN). To evaluate these models, we used data from the TCGA BC and normal samples, where 30% of the samples were randomly designated as the testing set, and the remaining samples served as the training set. Model performance was validated using 10-fold cross-validation with a fixed random seed of 42 to ensure reproducibility. We employed 6 key metrics to assess the diagnostic performance of the machine learning models: the area under the curve (AUC), sensitivity, specificity, positive predictive value (PPV), negative predictive value (NPV), and F1 score. These metrics provided a comprehensive evaluation of the predictive power and clinical relevance of the models.

### 2.3 Validation and clinical application of the ML diagnostic model

After evaluating and identifying the optimal ML diagnostic model using the TCGA dataset, we employed the GEO dataset as an independent external validation cohort to assess model generalizability. A similar evaluation strategy was used as in TCGA: the GEO samples were randomly split into 70% training and 30% testing subsets, and 10-fold cross-validation was performed within the 70% training set. The model parameters from the TCGA training cohort were directly applied without re-optimization, ensuring that this evaluation reflected true external validation. The classification performance of the ML model was further evaluated and visualized using a confusion matrix. Calibration curves were employed to assess the agreement between the model’s predicted probabilities and the actual outcomes, ensuring the reliability of its predictions. Decision Curve Analysis (DCA) was conducted to determine the clinical utility and net benefit of the model in real-world settings. Furthermore, the significance of individual features within the model was elucidated using SHapley Additive exPlanations (SHAP) values, derived through the “shap” software package. Force plots were generated to provide a detailed explanation of two representative cases, illustrating the contributions of different variables to the model’s predictions. The clinical applicability of the diagnostic model was also explored by evaluating its ability to diagnose BC across various clinical stages and PAM50 molecular subtypes using the TCGA dataset. These analyses highlight the model’s potential as a valuable tool for improving diagnostic accuracy and informing clinical decision-making in BC management.

### 2.4 Construction, evaluation, and validation of the ML prognostic model

After identifying candidate NRGs through univariate and multivariate Cox regression analyses, we employed the ‘glmnet’ package to fit a Lasso-Cox regression model. Gene expression and survival data were structured into a matrix format, and ten-fold cross-validation was used to determine the optimal penalty parameter (lambda). Features that were significantly associated with survival time in the model corresponded to non-zero regression coefficients. By extracting these non-zero coefficients, we identified NRGs that were significantly linked to OS. The risk score for each sample was calculated using the following formula: Risk score = (Coef_1_ × mRNA_1_ expression) + (Coef_2_ × mRNA_2_ expression) +… + (Coef_n_ × mRNA_n_ expression). Here, “Coef” represents the regression coefficient of each mRNA, derived through LASSO regression analysis. We stratified BC patients into high-risk and low-risk categories according to the median risk score. To explore the principal component analysis (PCA) features and t-distributed stochastic neighbor embedding (t-SNE) characteristics, we utilized the R packages “Rtsne” and “ggplot2”. The prognostic disparities between the two groups were meticulously analyzed using Kaplan-Meier (KM) survival analysis and the log-rank test. We utilized the “survival” and “timeROC” packages to conduct time-related receiver operating characteristic (ROC) analyses, evaluating the model’s predictive accuracy for 1-year, 3-year, and 5-year OS rates. Validation of these analyses was subsequently performed using the GEO external validation cohort. Furthermore, we explored the differences in risk scores among different clinical subgroups of BC, alongside examining the prognostic disparities between high-risk and low-risk groups within different clinical subgroups, to further evaluate the clinical relevance and generalizability of the model.

### 2.5 Construction and validation of a nomogram prognostic model based on risk scores and clinical characteristics

To ascertain whether the risk scores could function as an independent prognostic indicator for predicting patient OS, we integrated the risk scores with patient clinical characteristics into both univariate and multivariate regression analyses within the TCGA training cohort and the GEO validation cohort. Subsequently, leveraging the risk scores and clinical characteristics, we employed the “rms” package in R to develop nomograms that predict 1-year, 3-year, and 5-year OS. We evaluated the precision of these models through the generation of calibration curves and the execution of time-related ROC analyses. In the GEO validation cohort, we applied the same analytical framework to construct and evaluate the nomogram models, serving as a validation.

### 2.6 Functional enrichment analysis

In the TCGA cohort, we utilized the limma package to identify DEGs between high-risk and low-risk groups. The selection criteria for DEGs were defined as |Log Fold Change| > 1 and an adjusted P-value < 0.05. Subsequently, functional enrichment analyses, including Gene Ontology (GO) and Kyoto Encyclopedia of Genes and Genomes (KEGG) pathway enrichment, were performed using the “clusterProfiler” and “org.Hs.eg.db” R packages. Additionally, we conducted GO and KEGG analysis on differentially expressed NRGs between BC samples and normal samples. Furthermore, we employed Gene Set Enrichment Analysis (GSEA) to investigate pathway enrichment and biological differences between the two risk groups. To complement these findings, we conducted Disease Ontology (DO) analysis using the “DOSE” R package, identifying disease-associated biological processes and pathways linked to the identified DEGs.

### 2.7 TME analysis

To explore the differences in cancer hallmarks and their relevance to cell death pathways between high-risk and low-risk groups, we obtained relevant gene sets from previous studies (15, 16). Using the “GSVA” and “GSEABase” R packages, we performed single-sample gene set enrichment analysis (ssGSEA) to calculate enrichment scores for each gene set in each sample and analyzed their correlation with the risk scores. To further investigate differences in the immune microenvironment between the two groups, we identified markers associated with 16 types of immune cell infiltration and 13 immune functions (17). The ssGSEA algorithm was used to quantify the sample scores for these markers. Additionally, the immune, stromal, and ESTIMATE scores were calculated for each sample using the “estimate” R package. Xu et al. developed an online resource providing curated gene sets related to cancer progression and immune responses (18) (http://biocc.hrbmu.edu.cn/TIP/). Using gene sets obtained from this platform, we performed ssGSEA to evaluate their enrichment levels. Moreover, we retrieved expression data for major histocompatibility complex (MHC) molecules, chemokines and their receptors, and immune checkpoint genes (ICGs) from TCGA. Based on these datasets, we compared enrichment scores and gene expression levels between the two groups to assess variations in the TME.

### 2.8 Immunotherapy response prediction and drug sensitivity analysis

The Immunophenoscore (IPS) algorithm is a ML method used to predict the likelihood of response to cancer immunotherapy, specifically immune checkpoint inhibitors (19). We retrieved IIPS for BC samples from TCGA via The Cancer Immunome Atlas (TCIA) database. In addition, we employed the Tumor Immune Dysfunction and Exclusion (TIDE) algorithm to predict responses to immune therapy in these samples (20). To validate these immune therapy responses, we utilized data from the IMvigor210 study, which is based on a real-world patient cohort (21). Furthermore, using the Drug Sensitivity in Cancer (GDSC) database (22), we computed the 50% inhibitory concentration (IC50) values for 235 drugs against the BC samples, employing the “pRRophetic” R package. We conducted a correlation analysis between the IC50 values of each drug and the associated risk scores, identifying the top five drugs with positive and negative correlations to the risk scores. We then examined the differences in drug sensitivity between two defined risk groups, categorizing the samples into drug-sensitive and -insensitive groups based on the median IC50 values. The discriminative power of the risk scores to segregate these groups was assessed using ROC analysis. Moreover, we evaluated the efficacy of neoadjuvant chemotherapy across different risk groups of BC with data from GEO datasets GSE4779 and GSE25066.

### 2.9 Mutation analysis

We downloaded somatic mutation data for breast cancer samples from TCGA and utilized the “maftools” R package to create waterfall plots, which illustrated the mutational landscape in groups with high and low risk. Additionally, we calculated the tumor mutational burden (TMB) scores for these samples. Microsatellite instability (MSI) scores for the BC samples were acquired from a prior study (23). Furthermore, we computed intratumor heterogeneity (ITH) scores for each sample using the “DEPTH” package. We then analyzed the correlations between TMB, MSI, and ITH scores with risk scores, and assessed the differences in these metrics between the two risk groups.

### 2.10 Single cell, differential expression, and prognosis analyses of the NRGs for ML models construction

To delve deeper into the expression patterns of NRGs within the TME, we leveraged the Tumor Immune Single-cell Hub (TISCH) database (24) for a single-cell analysis, utilizing the GSE114727_10X dataset. Furthermore, we conducted a comparative analysis of the differential expression of NRGs between BC tissue and normal tissue samples, employing ROC curve analysis to evaluate their potential diagnostic utility. Additionally, we utilized KM survival analysis to elucidate the association between the expression levels of these NRGs and OS. This comprehensive approach not only augments our insight into the cellular heterogeneity of the TME but also underscores the pivotal role of NRGs as potential biomarkers in BC diagnostics and prognostics.

### 2.11 Blood samples collection, cell lines culture and quantitative real-time PCR

We obtained blood samples from 6 patients with breast fibroadenoma and 9 patients with BC who were treated at the Cancer Hospital of Shantou University Medical College. All patients were newly diagnosed and had not received any prior treatment. The final pathological diagnosis was confirmed through either core needle biopsy or surgical excisional biopsy.

The BC cell lines MCF-7 and MDA-MB-231, as well as the breast epithelial cell line MCF-10A, were purchased from Procell (Wuhan, China). They were cultured according to the supplier’s instructions.

Total RNA was extracted from these cells and blood samples using the RNAsimple Total RNA Kit (Tiangen, Beijing, China), following the manufacturer’s guidelines. Subsequently, qRT-PCR was performed using the PrimeScript™ RT Reagent Kit (Takara, Japan) and SYBR Premix Ex Taq™ II (Takara, Japan), adhering strictly to the manufacturer’s protocols. GAPDH was selected as the internal reference gene, and relative expression levels were calculated using the 2^−ΔΔ^Ct method. Two siRNAs were designed and selected based on the ULBP2 mRNA sequence for transfection into MDA-MB-231 cells. The knockdown efficiency was assessed by qRT-PCR after transfection. The specific primers used in this study are listed in **Supplementary Table S2**.

### 2.12 Cell viability assay (CCK8)

Transfected MDA-MB-231 cells were seeded into a 96-well plate at a density of 2,000 cells per well. Each group included three replicate samples, and the experiment was conducted multiple times. At 0, 24, 48, and 72 hours post-seeding, 10 µL of CCK-8 reagent was added to each well, followed by a 2-hour incubation. Optical density (OD) at a wavelength of 450 nm was measured using a spectrophotometer. Growth curves were generated and cell viability for each group was calculated.

### 2.13 Clone formation assay

Transfected MDA-MB-231 cells were seeded at a density of 1,000 cells per well in a six-well plate and incubated in a CO2 incubator for 14 days. The medium was refreshed every 2–3 days, and clone formation was monitored. Once clones formed, cells were fixed with 4% paraformaldehyde for 30 minutes, stained with 0.1% crystal violet for 20 minutes, air-dried, photographed, and images were recorded.

### 2.14 Transwell invasion and migration assay

After a 12-hour starvation period, transfected MDA-MB-231 cells were trypsinized and resuspended at a concentration of 4×10^^4^ cells per mL in serum-free medium. For the migration assay, 300 µL of the cell suspension was placed in the upper chamber, and the lower chamber was filled with 600 µL of medium containing 20% fetal bovine serum. The invasion assay included an initial step of coating the upper chamber with 100 µL of diluted Matrigel, which was allowed to solidify at 37°C for 2 hours. Subsequently, the cell suspension was added, and both assays were conducted for 24 to 48 hours. After the incubation period, the chambers were washed with PBS at room temperature, fixed with 4% paraformaldehyde for 30 minutes, and stained with 0.1% crystal violet for 20 minutes. After drying, images were captured at room temperature using an inverted microscope and saved for analysis.

### 2.15 Statistical analysis

Statistical analysis was conducted with R software (version 4.0.3) or Python (version 3.8). The Wilcoxon signed-rank test was applied to evaluate differences in continuous variables between two groups, while the Kruskal-Wallis test was used for comparisons across more than two groups. For categorical variables, chi-square tests were employed. Correlations were assessed using Spearman’s rank correlation. A p-value below 0.05 was considered statistically significant.

## 3 Result

### 3.1 Candidate NRGs screening for ML models construction

**Figure 2:**
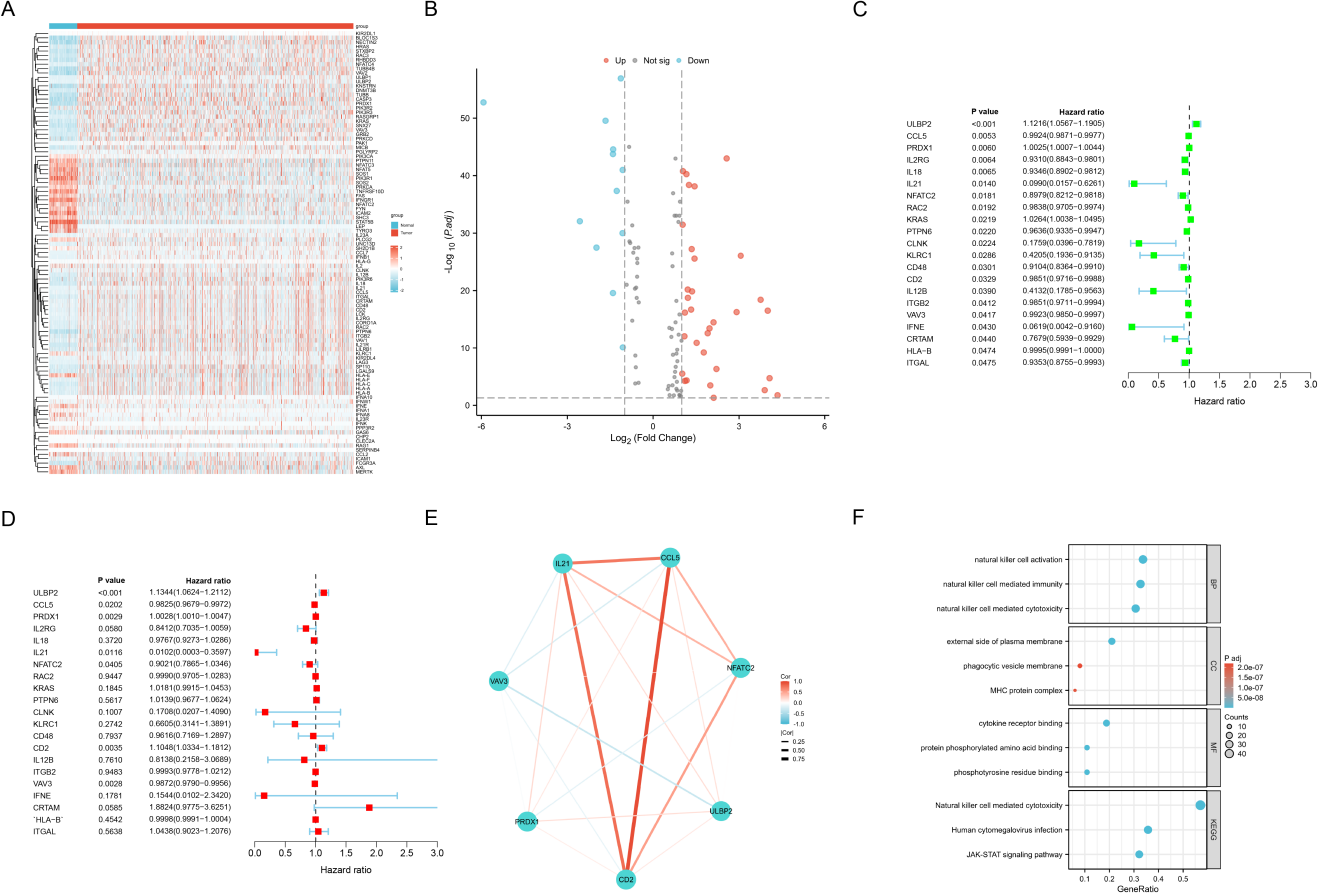
Candidate NRGs screening for ML models construction. **(A)** Heat map shows differentially expressed NRGs expression between the BC and normal groups from TCGA, with red indicating up-regulation and blue indicating down-regulation. **(B)** Volcano plot shows differentially expressed NRGs expression between the BC and normal groups with |Log Fold Change| > 1 and adjusted P-value < 0.05. **(C, D)** Univariate **(C)** and multivariate **(D)** Cox regression analyses to identify seven NRGs most strongly associated with OS. **(E)** Network diagram illustrates the Spearman correlations among the identified NRGs. **(F)** Bubble plot illustrates the key findings of the GO and KEGG pathway enrichment analyses for differentially expressed NRGs between the BC and normal groups.

As shown in **Supplementary Table S3**, we identified 101 differentially expressed NRGs, of which 33 were down-regulated and 68 were up-regulated in BC. The expression profiles of these NRGs between the BC and normal sample groups were visualized using heatmaps (**Figure 2A**) and volcano plots (**Figure 2B**). Subsequently, through univariate (**Figure 2C**) and multivariate Cox regression analyses (**Figure 2D**), we identified seven NRGs most strongly associated with OS (p<0.05), namely ULBP2, CCL5, PRDX1, IL21, NFATC2, CD2, and VAV3. A correlation network diagram was constructed to illustrate the Spearman correlations among these seven NRGs (**Figure 2E**), with the strongest positive correlations observed between CD2, CCL5, and IL21. Furthermore, based on these 101 differentially expressed NRGs, we performed GO and KEGG pathway enrichment analyses, with the results provided in **Supplementary Table S4**. The key findings were visualized using a bubble plot (**Figure 2F**), revealing that the enriched pathways primarily involved immune responses, cytokine signaling, immune evasion, cell membrane functions, and signal transduction, suggesting a crucial role for NRGs in the TME.

### 3.2 Construction and evaluation of 12 ML diagnostic models

**Figure 3:**
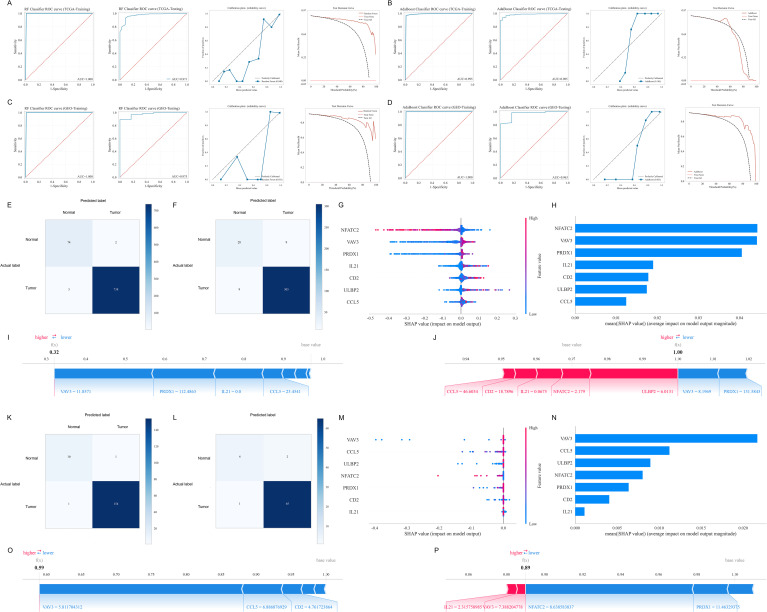
Construction, evaluation, interpretability and validation of the ML diagnostic models. **(A, B)** ROC curves, calibration plots, and test decision curves of RF **(A)** and AdaBoost **(B)** models in the TCGA training cohort. **(C, D)** ROC curves, calibration plots, and test decision curves of RF **(C)** and AdaBoost **(D)** models in the GEO validation cohort. **(E, F)** Confusion matrices of the RF model in the training set **(E)** and testing set **(F)** of the TCGA cohort. **(G, H)** SHAP values for each feature at different levels **(G)** and important features **(H)** of the RF model in the TCGA cohort. **(I, J)** Interpretability of the RF model with a representative sample whose actual and predicted outcomes are both normal **(I)** and a representative sample whose actual and predicted outcomes are both BC **(J)** in the TCGA cohort. **(K, L)** Confusion matrices of the RF model in the training set **(K)** and testing set **(L)** of the GEO cohort. **(M, N)** SHAP values for each feature at different levels **(M)** and important features **(N)** of the RF model in the GEO cohort. **(O, P)** Interpretability of the RF model with a representative sample whose actual and predicted outcomes are both normal **(O)** and a representative sample whose actual and predicted outcomes are both BC **(P)** in the GEO cohort.

After identifying the candidate NRGs and feature selection, all seven NRGs were incorporated into the ML diagnostic models construction (**Supplementary Figure S1**). We utilized 12 ML algorithms to construct diagnostic models for BC, with the performance of each model on both the training and testing sets summarized in **Table 1**. These results indicate that, within the TCGA training cohort, the RF model demonstrated exceptional accuracy and reliability on the training set, whereas the AdaBoost model stood out in several critical metrics, emerging as the optimal model for the testing set. In the TCGA training cohort, the RF model achieved an AUC of 1.0 on the training set, and an AUC of 0.971 on the testing set. The RF model exhibited smaller calibration errors compared to the AdaBoost model and showed superior performance in the test decision curve (**Figures 3A, B**). However, the AdaBoost model exhibited higher AUC, sensitivity, specificity, and other metrics on the testing set. To further validate these findings, we evaluated the performance of both models in the GEO validation cohort. The results showed that, regardless of whether in the training or testing set, the RF model consistently yielded higher AUC (**Figures 3C, D**). Although the AdaBoost model performed comparably well, the RF model demonstrated more consistent predictive ability across cross-validation folds, better external validation generalization, making it a more suitable choice for our diagnostic application. Thus, we ultimately selected the RF model as the most optimal diagnostic model based on its superior performance.

**Table 1:** Performance of ML diagnostic models in training and testing sets.

Sets	Models	AUC	Accuracy	Sensitivity	Specificity	PPV	NPV	F1‐score
Training set	LR	0.970	0.893	0.887	0.952	0.994	0.466	0.937
	XGBoost	0.986	0.941	0.938	0.971	0.997	0.616	0.966
	LightGBM	0.840	0.240	0.168	0.950	NaN	0.139	NaN
	RF	1.000	0.999	0.999	1.000	1.000	0.986	0.999
	AdaBoost	0.995	0.970	0.968	0.997	1.000	0.762	0.983
	DT	0.975	0.767	0.744	0.988	NaN	0.348	NaN
	GB	1.000	0.998	0.998	1.000	1.000	0.980	0.999
	GNB	0.957	0.899	0.894	0.948	0.994	0.493	0.941
	CNB	0.909	0.821	0.818	0.853	0.981	0.343	0.892
	MLP	0.578	0.548	0.531	0.703	0.949	0.151	0.655
	SVM	0.953	0.900	0.901	0.892	0.988	0.501	0.942
	KNN	1.000	0.097	0.000	1.000	NaN	0.097	NaN
Testing set	LR	0.963	0.912	0.914	0.892	0.986	0.55	0.949
	XGBoost	0.923	0.929	0.936	0.865	0.983	0.615	0.919
	LightGBM	0.831	0.835	0.844	0.757	0.967	0.346	0.901
	RF	0.971	0.949	0.971	0.757	0.971	0.757	0.971
	AdaBoost	0.985	0.96	0.978	0.811	0.978	0.811	0.978
	DT	0.885	0.105	0.000	1.000	NaN	0.105	NaN
	GB	0.961	0.949	0.984	0.649	0.96	0.828	0.972
	GNB	0.929	0.892	0.912	0.706	0.967	0.462	0.939
	CNB	0.937	0.835	0.818	1.000	1.000	0.37	0.900
	MLP	0.431	0.841	0.918	0.118	0.907	0.133	0.913
	SVM	0.962	0.892	0.887	0.941	0.993	0.471	0.937
	KNN	0.937	0.097	0.000	1.000	NaN	0.097	NaN

### 3.3 Interpretability, validation and clinical application of the RF diagnostic model

We visualized the detailed comparison between the actual and predicted labels for both the training (**Figure 3E**) and testing sets (**Figure 3F**) in the TCGA cohort using confusion matrices, and further validated the results in the GEO cohort (**Figures 3K, L**). **Figures 3G, M** respectively show the SHAP values for each feature at different levels in the TCGA training cohort and GEO validation cohort. As the feature value increases, the color gradually shifts to red, whereas lower values correspond to a blue color. Additionally, we ranked the features based on their importance (**Figures 3H, N**). A higher rank indicates greater importance, meaning the feature contributes more to the model’s predictions. In the TCGA cohort, the NRGs contributing most to the RF model were primarily NFATC2, VAV3, and PRDX1, while in the GEO cohort, VAV3 was the most significant. We further illustrated the interpretability of the RF model by showcasing representative samples. In the TCGA cohort, a normal sample had a relatively low SHAP prediction score of 0.32 (**Figure 3I**), while a BC sample had a higher SHAP prediction score of 1.00 (**Figure 3J**). Similarly, two representative samples were selected and validated in the GEO cohort (**Figures 3O, P**). Furthermore, we obtained blood samples from 6 patients with breast fibroadenoma and 9 patients with BC as a clinical validation cohort for the diagnostic model. The RF model demonstrated robust performance in this cohort, achieving an AUC of 0.811 (**Supplementary Figure S2A**), and DCA confirmed its clinical applicability (**Supplementary Figure S2B**).

Additionally, we evaluated the clinical application value of the RF model. As shown in **Supplementary Figure S3**, the RF diagnostic model demonstrated an AUC close to 1.0 in both the training and testing sets across different pathologic stages and PAM50 subtypes of BC. This highlights the high accuracy and universality of the ML diagnostic model, showcasing its promising performance and potential for clinical application.

### 3.4 Construction, evaluation and validation of the ML prognostic model

**Figure 4:**
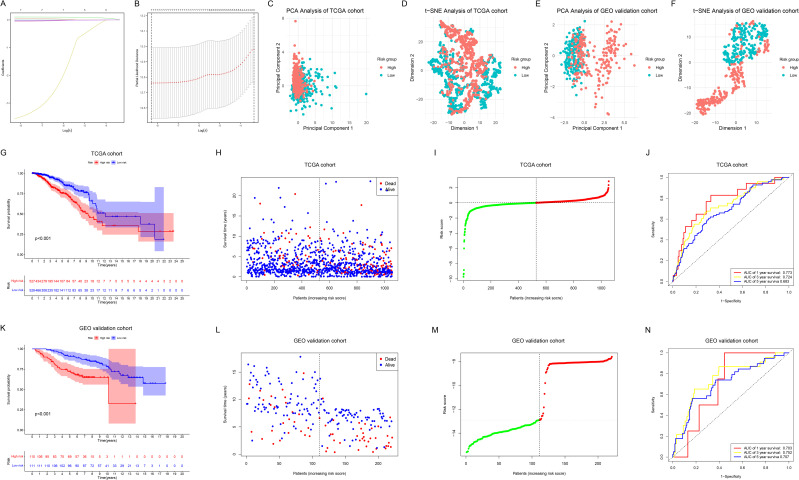
Construction, evaluation and validation of the ML prognostic model. **(A)** LASSO coefficient profiles of 7 prognostic NRGs. **(B)** The outcomes of ten-fold cross-validation indicated the optimal value of the penalty parameter. 7 independent prognostic NRGs were selected to construct the risk model. **(C, D)** PCA **(C)** and t-SNE **(D)** analyses between the two risk groups in the TCGA cohort. **(E, F)** PCA **(E)** and t-SNE **(F)** analyses between the two risk groups in the GEO cohort. **(G)** KM survival analysis between the two risk groups in the TCGA cohort. **(H, I)** Survival status **(H)** and risk score distribution **(I)** of the patients in the TCGA cohort. **(J)** ROC curves for predicting 1-, 3-, and 5-year OS in the TCGA cohort. **(K)** KM survival analysis between the two risk groups in the GEO cohort. **(L, M)** Survival status **(L)** and risk score distribution **(M)** of the patients in the GEO cohort. **(N)** ROC curves for predicting 1-, 3-, and 5-year OS in the GEO cohort.

After identifying the candidate NRGs, we constructed a prognostic model using LASSO regression analysis (**Figures 4A, B**). The final risk score for each sample was calculated using the following formula (Equation 1):


(1) $$\begin{array}{c} \text{Risk score}=0.106\times ULBP2-0.019\times CCL5+0.003\\ \times PRDX1-3.561\times IL21-0.048\times NFATC2\\ +0.055\times CD2-0.009\times VAV3 \end{array}$$

PCA (**Figure 4C**) and t-SNE (**Figure 4D**) analyses revealed distinct clustering between the low-risk and high-risk groups in the TCGA cohort, which was further validated in the GEO cohort (**Figures 4E, F**). KM survival analysis (**Figure 4G**) demonstrated that OS was significantly shorter in the high-risk group than in the low-risk group in the TCGA cohort (p < 0.001). **Figures 4H, I** respectively illustrate the survival status and risk score distribution of the patients. Time-dependent ROC analysis indicated that the model’s AUC for predicting 1-year, 3-year, and 5-year OS was 0.773, 0.724, and 0.683, respectively (**Figure 4J**). To assess the model’s applicability and reliability, we applied the above formula to calculate the risk scores for each BC sample in the GEO external validation cohort, successfully validating our findings (**Figures 4K–N**).

### 3.5 Clinical relevance of the risk scores and clinical subgroups analysis

The clinical information of BC patients from TCGA is summarized in **Table 2**. We grouped the patients based on age, T stage, N stage, M stage, pathological stage, estrogen receptor (ER) status, progesterone receptor (PR) status, human epidermal growth factor receptor 2 (HER2) status, PAM50 subtype, and survival status, and analyzed the differences in risk scores among the subgroups. As shown in **Supplementary Figure S4**, we observed an increasing trend in the risk score in patients with pathological stage IV, and it was higher in the M1 stage compared to the M0 stage (p<0.05), suggesting that the risk score effectively reflects the severity of the disease, particularly in relation to features associated with distant metastasis. Moreover, we found that BC patients who were ER-negative and PR-negative had higher risk scores (both p<0.001), while patients with Luminal A subtype had lower risk scores compared to those with Luminal B, HER2-enriched, and Basal-like subtypes (all p<0.05).

**Table 2:** Clinical characteristics of BC patients from TCGA.

Clinical characteristics	Group	No. of case (%)
Age (year)	<60	588 (53.73)
	≥60	467 (46.27)
T stage	T1	275 (26.07)
	T2	610 (57.82)
	T3	134 (12.70)
	T4	33 (3.13)
	Unknown	3 (0.28)
N stage	N0	499 (47.30)
	N1	347 (32.89)
	N2	116 (11.0)
	N3	74 (7.01)
	Unknown	19 (1.80)
M stage	M0	879 (83.32)
	M1	20 (1.90)
	Unknown	156 (14.79)
Pathologic stage	I	180 (17.06)
	II	597 (56.59)
	III	236 (22.37)
	IV	18 (1.71)
	Unknown	24 (2.27)
ER status	Positive	770 (72.99)
	Negative	237 (22.46)
	Unknown	48 (4.55)
PR status	Positive	670 (63.51)
	Negative	334 (31.66)
	Unknown	51 (4.83)
HER2 status	Positive	153 (14.50)
	Negative	544 (51.56)
	Unknown	358 (33.93)
Subtype	Normal-like	35 (3.32)
	Luminal A	490 (46.45)
	Luminal B	192 (18.20)
	HER2-enriched	75 (7.11)
	Basal-like	169 (16.02)
	Unknown	94 (8.91)
Survival status	Alive	908 (86.07)
	Dead	147 (13.93)

Additionally, we analyzed the OS differences between high-risk and low-risk patients within each clinical subgroup. In the majority of clinical subgroups, high-risk patients had significantly poorer prognoses compared to the low-risk group (all p<0.05), although no statistical difference was observed in M1-stage patients, as well as those with Normal-like, Luminal A, Luminal B, and HER2-enriched subtypes (p>0.05) (**Supplementary Figure S5**).

### 3.6 Construction and validation of the nomogram prognostic model

**Figure 5:**
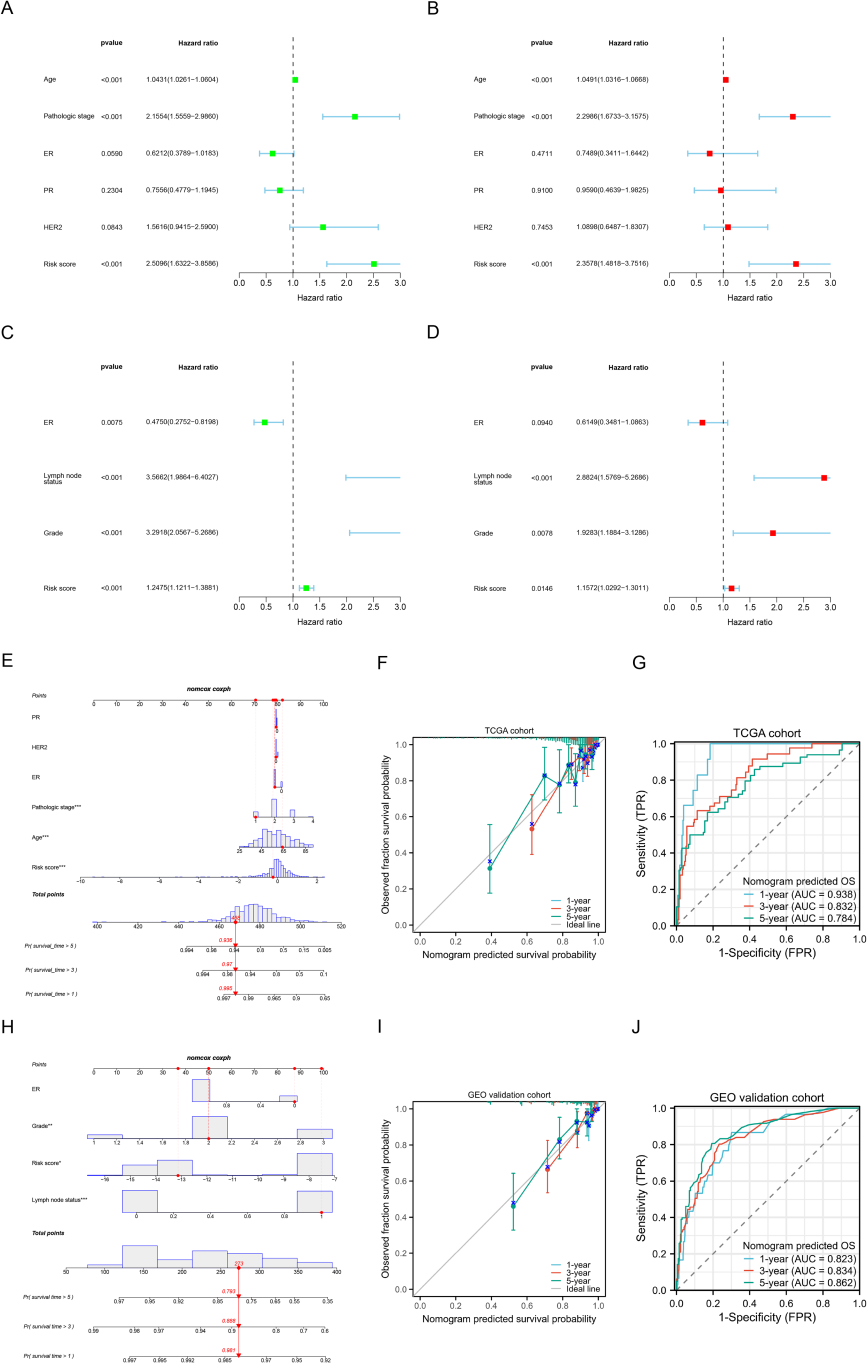
Construction and validation of nomogram prognostic models. **(A, B)** Univariate **(A)** and multivariate **(B)** Cox regression analysis of the risk scores and clinical characteristics in the TCGA training cohort. **(C, D)** Univariate **(C)** and multivariate **(D)** Cox regression analysis of the risk scores and clinical characteristics in the GEO validation cohort. **(E)** Nomogram prognostic model for predicting the 1-, 3- and 5-year OS probabilities in the TCGA cohort. **(F)** Calibration curve of the nomogram to predict 1-, 3- and 5-year OS probabilities in the TCGA cohort. **(G)** Time-dependent ROC curves of the nomogram to predict 1-, 3- and 5-year OS probabilities in the TCGA cohort. **(H)** Nomogram prognostic model for predicting the 1-, 3- and 5-year OS probabilities in the GEO cohort. **(I)** Calibration curve of the nomogram to predict 1-, 3- and 5-year OS probabilities in the GEO cohort. **(J)** Time-dependent ROC curves of the nomogram to predict 1-, 3- and 5-year OS probabilities in the GEO cohort. *P < 0.05, **P < 0.01, ***P < 0.001.

In the TCGA cohort, we included age, pathological stage, ER, PR, and HER2 status in both univariate and multivariate regression analyses. Univariate analysis (**Figure 5A**) revealed that age, pathological stage, and risk scores were associated with OS (all p<0.05). Multivariate regression analysis (**Figure 5B**) indicated that the risk scores is an independent prognostic factor for predicting OS in BC patients (p<0.001). In the GEO validation cohort, the risk scores was also identified as an independent prognostic factor for OS (p<0.05) (**Figures 5C, D**). The clinical information for the GEO cohort is provided in **Supplementary Table S5**. Subsequently, by combining the risk scores with patient clinical characteristics, we constructed a Nomogram prognostic model to predict 1-year, 3-year, and 5-year OS (**Figure 5E**), and evaluated its accuracy using calibration curves (**Figure 5F**) and time-dependent ROC curves (**Figure 5G**). The time-dependent ROC analysis revealed that the AUCs for predicting 1-year, 3-year, and 5-year OS were 0.938, 0.832, and 0.784, respectively. We similarly constructed a nomogram prognostic model in the GEO cohort by combining the risk scores with clinical features (**Figure 5H**) and performed evaluations (**Figures 5I, J**), further demonstrating the predictive potential of the risk scores when combined with clinical indicators for prognosis.

### 3.7 Functional enrichment analysis

**Figure 6:**
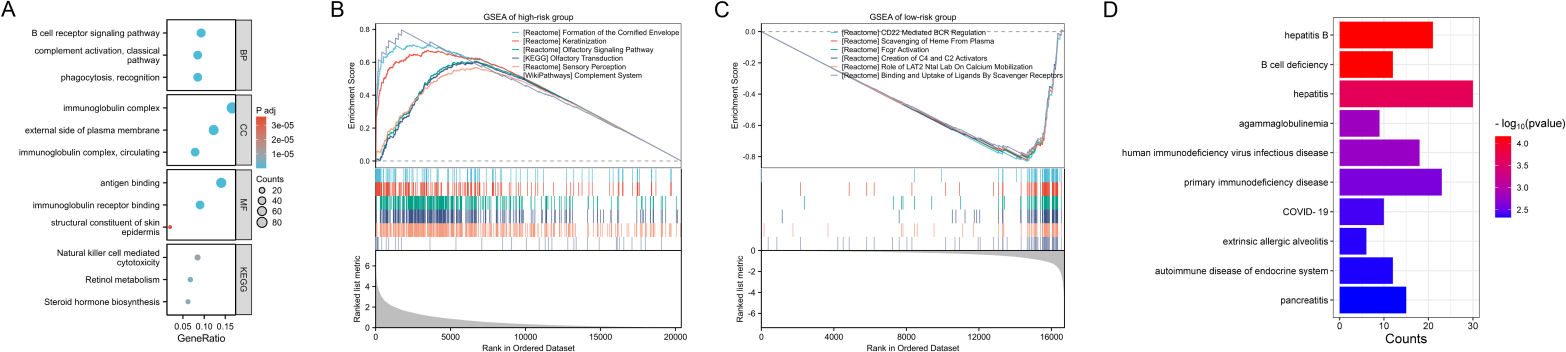
Functional enrichment analysis. **(A)** GO and KEGG pathway enrichment analyses on the DEGs between the two risk groups. **(B, C)** GSEA of the high-risk **(B)** and low-risk groups. **(D)** DO analysis on the DEGs between the two risk groups.

Between the two risk groups, we identified a total of 1369 DEGs (**Supplementary Table S6**), and performed GO and KEGG pathway enrichment analyses on these DEGs (**Supplementary Table S7**). The main findings, as shown in **Figure 6A**, suggest that immune responses, antigen recognition, cellular metabolism, and endocrine regulation may exhibit significant differences between the two risk groups, implying that the DEGs may play a critical role in tumorigenesis or immune-related diseases. We further conducted GSEA for the high-risk (**Supplementary Table S8**) and low-risk groups (**Supplementary Table S9**). The enriched pathways in the high-risk group were mainly associated with skin development, keratinization, olfactory perception, complement system, among others, all of which are linked to immune responses, cellular differentiation, and sensory functions. This suggests that the high-risk group may have a stronger response in immune activity, cellular metabolism, and microenvironment regulation (**Figure 6B**). In contrast, the enriched pathways in the low-risk group were predominantly involved in immune response-related pathways, including B cell receptor regulation, complement system, and scavenger receptors, indicating that the low-risk group may have a more robust immune surveillance function, with a prominent role of B cells and the complement system in immune responses (**Figure 6C**). These differences suggest that the high-risk group may exhibit more complex immune reactions and cellular environment alterations, potentially associated with tumor progression, while the low-risk group may rely on more stable immune surveillance mechanisms, exhibiting stronger immune responses. Additionally, we conducted DO analysis for the DEGs between the two groups (**Supplementary Table S10**). The diseases enriched in this analysis suggest that the high-risk group may exhibit characteristics such as immune dysfunction, immune evasion mechanisms, immune deficiencies, or hyperactive immune responses, particularly in immune deficiency diseases like B cell deficiency, primary immunodeficiencies, HIV infection, and immunoglobulin deficiencies (**Figure 6D**). These findings may indicate a weakened immune response in the high-risk group, making them more susceptible to infections or chronic immune diseases. At the same time, diseases related to immune-mediated inflammation, such as hepatitis, pancreatitis, and allergic alveolitis, may suggest that this group exhibits heightened immune activity or an overactive immune response.

### 3.8 Cancer hallmarks and cell death pathways analyses

We investigated the differences in cancer hallmarks within the tumor TME between the two risk groups and found that the high-risk group was primarily enriched in pathways such as the G2M checkpoint, tumor proliferation signature, DNA replication, MYC targets, and cellular response to hypoxia (**Supplementary Figure S6A**). Notably, these gene signatures showed the strongest positive correlation with the risk score (**Supplementary Figure S6B**), indicating that tumor cells in the high-risk group possess robust proliferative and adaptive capabilities, enabling them to survive and grow under stress conditions such as rapid proliferation, genomic instability, and hypoxia. These features are typically associated with tumor aggressiveness, metastatic potential, and resistance to therapy. In the cell death pathways, Oxeiptosis was primarily enriched in the high-risk group and showed the strongest positive correlation with the risk scores (**Supplementary Figures S6C, D**), while the low-risk group was predominantly enriched in pathways related to necroptosis, immunogenic cell death, and pyroptosis.

### 3.9 Immune characteristic analysis

**Figure 7:**
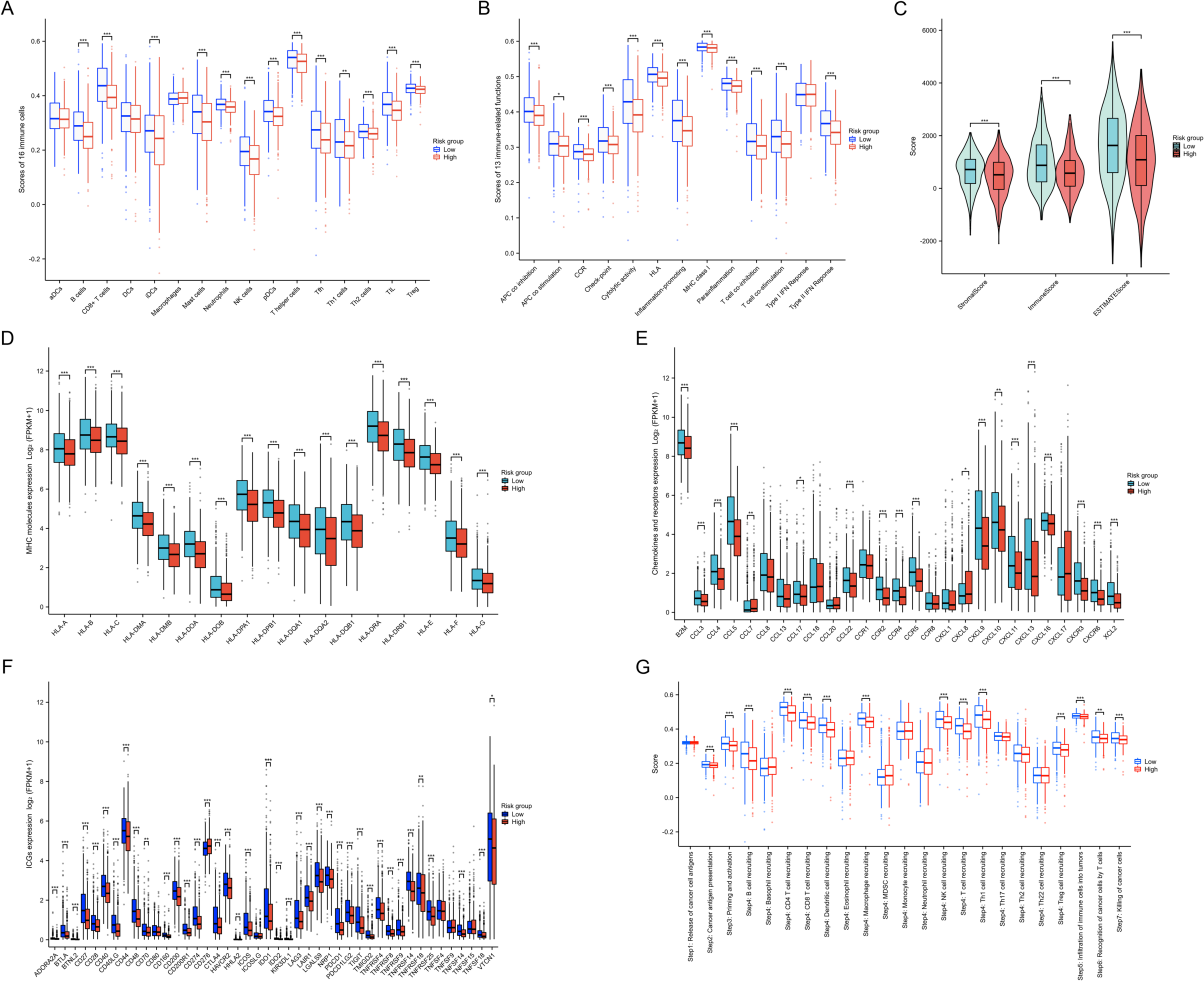
Immune characteristics between two risk groups. **(A)** Differences in 16 types of immune cell infiltration between the two risk groups. **(B)** Differences in 13 types of immune functions between the two risk groups. **(C)** Differences in immune, stromal, and ESTIMATE scores between the two risk groups. **(D)** Differences in MHC molecules expression between the two risk groups. **(E)** Differences in chemokines and receptors expression between the two risk groups. **(F)** Differences in ICG expression between the two risk groups. **(G)** Differences in enrichment scores of gene sets related to cancer progression and immune responses between the two risk groups. *P < 0.05, **P < 0.01, ***P < 0.001.

By analyzing the immune characteristic within the TME, we found a reduction in the infiltration of anti-tumor immune cells in the high-risk group, such as CD8+ T cells, NK cells, tumor-infiltrating lymphocytes, CD4+ T cells, and T follicular helper cells (all p<0.001) (**Figure 7A**). In the high-risk group, most immune functions were down-regulated, including APC co-inhibition, CC chemokine receptors, checkpoint regulation, cytolytic activity, human leukocyte antigen, inflammation-promoting factors, MHC class I molecules, parainflammation, T cell co-inhibition, T cell co-stimulation, and Type II IFN response (all p<0.001) (**Figure 7B**). Moreover, immune scores, stromal scores, and ESTIMATE scores were significantly lower in the high-risk group compared to the low-risk group (all p<0.001) (**Figure 7C**). Additionally, the expression of the majority of MHC molecules (**Figure 7D**), chemokines and receptors (**Figure 7E**), and ICGs (**Figure 7F**) was suppressed in the high-risk group (p<0.05). By analyzing the differences in anti-tumor immune responses across multiple steps between the two groups, we observed a marked suppression of immune responses in the high-risk group (p<0.01) (**Figure 7G**). These results reveal an enhanced tumor immune escape mechanism in the high-risk group, as well as a weakened immune surveillance function. This suggests that tumors in the high-risk group are more likely to evade detection and elimination by the host immune system, leading to a poorer prognosis.

### 3.10 Immunotherapy response prediction

The IPS score, which evaluates the composition and functional status of immune cells in the TME, helps predict patient responses to immune checkpoint inhibitors. We found that regardless of PD-1 and CTLA-4 expression status, the IPS score was significantly lower in the high-risk group (all p<0.001) (**Supplementary Figure S7A**). While there were no differences in TIDE scores between the two groups (p>0.05), the high-risk group exhibited lower expression of IFNG, Merck18, CD274, CD8, among others, along with a lower Dysfunction score and a higher Exclusion score (all p<0.001) (**Supplementary Figure S7B**), suggesting a stronger immune escape mechanism in high-risk BC patients. Additionally, in the IMvigor210 real-world study cohort, the low-risk group showed a higher response rate to immune therapy (p<0.01), although no statistical difference was observed in the TCGA cohort (p=0.52) (**Supplementary Figures S7C, D**). Overall, these findings emphasize that high-risk patients may exhibit more robust immune escape features, which could result in a poorer response to immune checkpoint inhibitors. Therefore, evaluating the IPS score and immune escape mechanisms may help predict which patients are more likely to benefit from immunotherapy.

### 3.11 Drug sensitivity analysis

**Figure 8:**
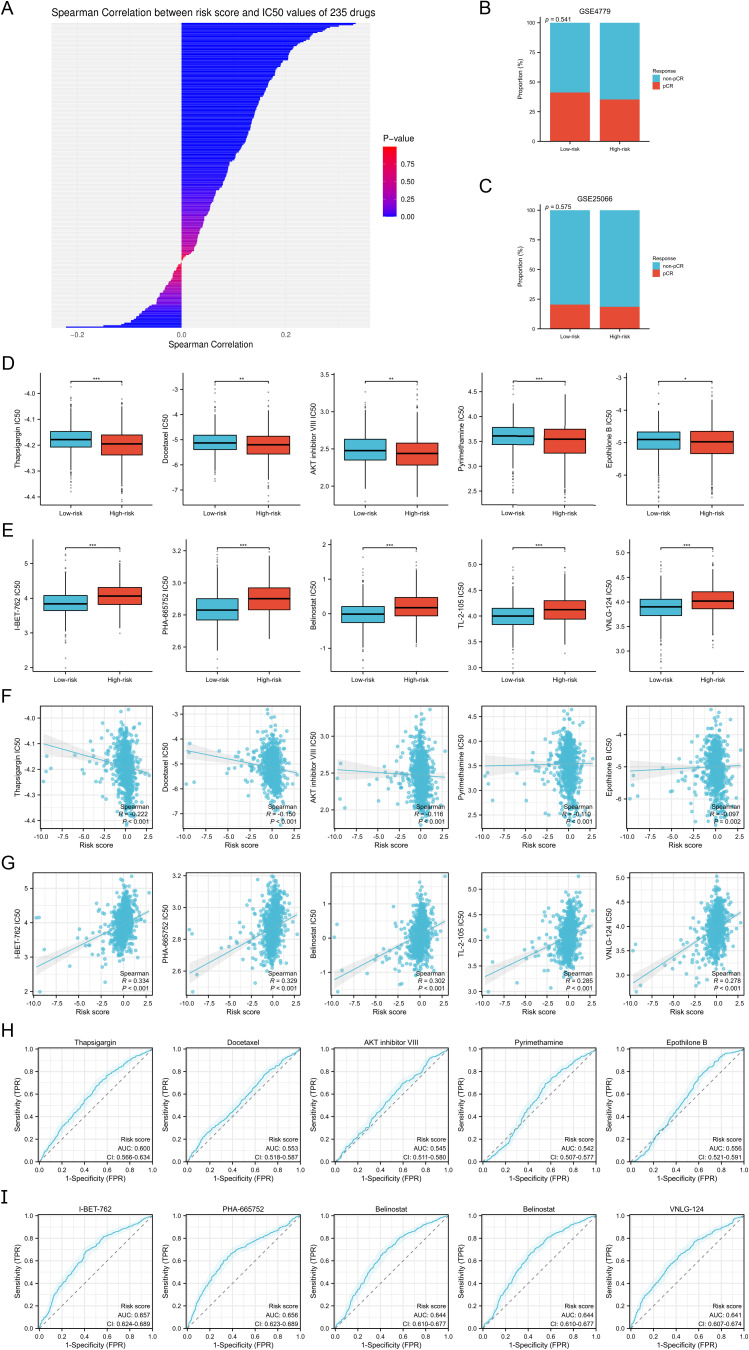
Drug sensitivity analysis. **(A)** Spearman correlation analysis between the risk scores and IC50 values of 235 drugs. **(B, C)** Proportion of BC patients who achieved pCR and non-pCR after neoadjuvant chemotherapy in the GSE4779 **(B)** and GSE25066 **(C)** cohorts. **(D, E)** Differences in IC50 values of the top five drugs negatively **(D)** and positively **(E)** correlate with the risk scores between the two risk groups. **(F, G)** Spearman correlation analysis between the risk scores and IC50 values of the top five drugs negatively **(F)** and positively **(G)** correlate with the risk scores. **(H, I)** ROC analysis to evaluate the discriminative power of the risk scores in the drug-sensitive and drug-insensitive groups of the top five drugs negatively **(H)** and positively **(I)** correlate with the risk scores. *P < 0.05, **P < 0.01, ***P < 0.001.

Based on the GDSC database, we calculated the IC50 values of 235 drugs and assessed their correlation with the risk scores (**Supplementary Table S11**, **Figure 8A**). Additionally, through the GSE4779 and GSE25066 cohorts, we found that the proportion of BC patients in the high-risk group achieving pathological complete response (pCR) after neoadjuvant chemotherapy was lower, indicating a poorer response to neoadjuvant chemotherapy, although no statistical significance was reached (both p>0.05) (**Figures 8B, C**). Through drug sensitivity analysis, we identified the five drugs most strongly negatively correlated with risk scores, namely Thapsigargin, Docetaxel, AKT Inhibitor VIII, Pyrimethamine, and Epothilone B. Conversely, the five drugs most strongly positively correlated with risk scores were I-BET-762, PHA-665752, Belinostat, TL-2-105, and VNLG_124. We displayed the differences in the IC50 values of these drugs between the two risk groups using box plots (**Figures 8D, E**), and further illustrated the correlation between IC50 values and risk scores with scatter plots (**Figures 8F, G**). Additionally, patients were categorized into drug-sensitive and drug-insensitive groups based on the median IC50 values for each drug. Through ROC analysis, we found that among the top five drugs most strongly positively correlated with risk scores, the risk scores demonstrated the strongest ability to distinguish the drug-sensitive and drug-insensitive groups for Thapsigargin, with an AUC of 0.6 (**Figure 8H**). Among the top five drugs most strongly negatively correlated with risk scores, the risk scores exhibited the strongest ability to differentiate for I-BET-762 (**Figure 8I**). The drug sensitivity analysis highlights the risk scores as an important predictive factor, aiding in the identification of patient populations sensitive or resistant to specific drugs. This provides a theoretical foundation for personalized drug therapy in the treatment for BC.

### 3.12 Mutation analysis

We examined somatic mutation data from two risk groups, displaying the results via waterfall plots (**Supplementary Figures S8A, B**). In the low-risk group, PIK3CA mutations were the most common, occurring in 38% of cases. Conversely, TP53 mutations were the most frequent in the high-risk group, found in 44% of patients. Moreover, in both groups, single nucleotide variations (SNVs) were the predominant variation type, with missense mutations being the most frequent variant category (**Supplementary Figures S8C, D**). Furthermore, we observed that TMB and ITH scores were higher in the high-risk group and positively correlated with risk scores (all p<0.001), while MSI scores showed no significant correlation with risk scores (p>0.05) (**Supplementary Figures S8E–J**).

### 3.13 Single cell analysis

We performed single cell analysis using the GSE114727_10X dataset from the TISCH database to investigate the expression patterns of 7 NRGs in immune-related cells of the BC tumor microenvironment. The cell type annotations are shown in **Supplementary Figures S9A**, which include CD4+ T conventional cells, CD8+ T cells, CD8+ T effector memory cells, Tprolif, and regulatory T cells. These five cell types were further divided into 17 distinct cell populations (**Supplementary Figures S9B**). **Supplementary Figures S9C and S9D** present the quantities and proportions of different cell types in the GSE114727_10X dataset. Additionally, **Supplementary Figures S9E** displays the percentage and expression levels of the 7 NRGs. Among them, ULBP2, IL21, and VAV3 are almost negligibly expressed in the immune microenvironment. CCL5 exhibits strong expression in CD4+ regulatory T cells, CD8+ terminally differentiated T cells, and CD8+ T cells, while PRDX1 and CD2 show moderate expression across all five cell types. NFATC2 is expressed at low levels across various cell types.

### 3.14 Differential expression and survival analyses of the 7 NRGs

In the TCGA cohort, we analyzed the differential expression of the 7 NRGs used to construct the models in BC and normal tissues, and explored their diagnostic value for BC through ROC analysis. Among these NRGs, except for NFATC2, which was expressed at lower levels in BC compared to normal tissues, the remaining NRGs were highly expressed in BC tissues (all p<0.05) (**Supplementary Figures S9F**). Notably, PRDX1 demonstrated a superior diagnostic ability for BC, with an AUC of 0.864 (**Supplementary Figures S9G**). Furthermore, we validated the differential expression of these NRGs in cell lines using qRT-PCR (**Supplementary Figures S9H**). In the KM survival analysis, patients with high expression of ULBP2 had poorer DSS, while patients with high expression of CCL5 and CD2 had better OS (all p<0.05) (**Supplementary Figure S10**).

### 3.15 Knockdown of ULBP2 inhibits tumor cell proliferation, migration and invasion

**Figure 9:**
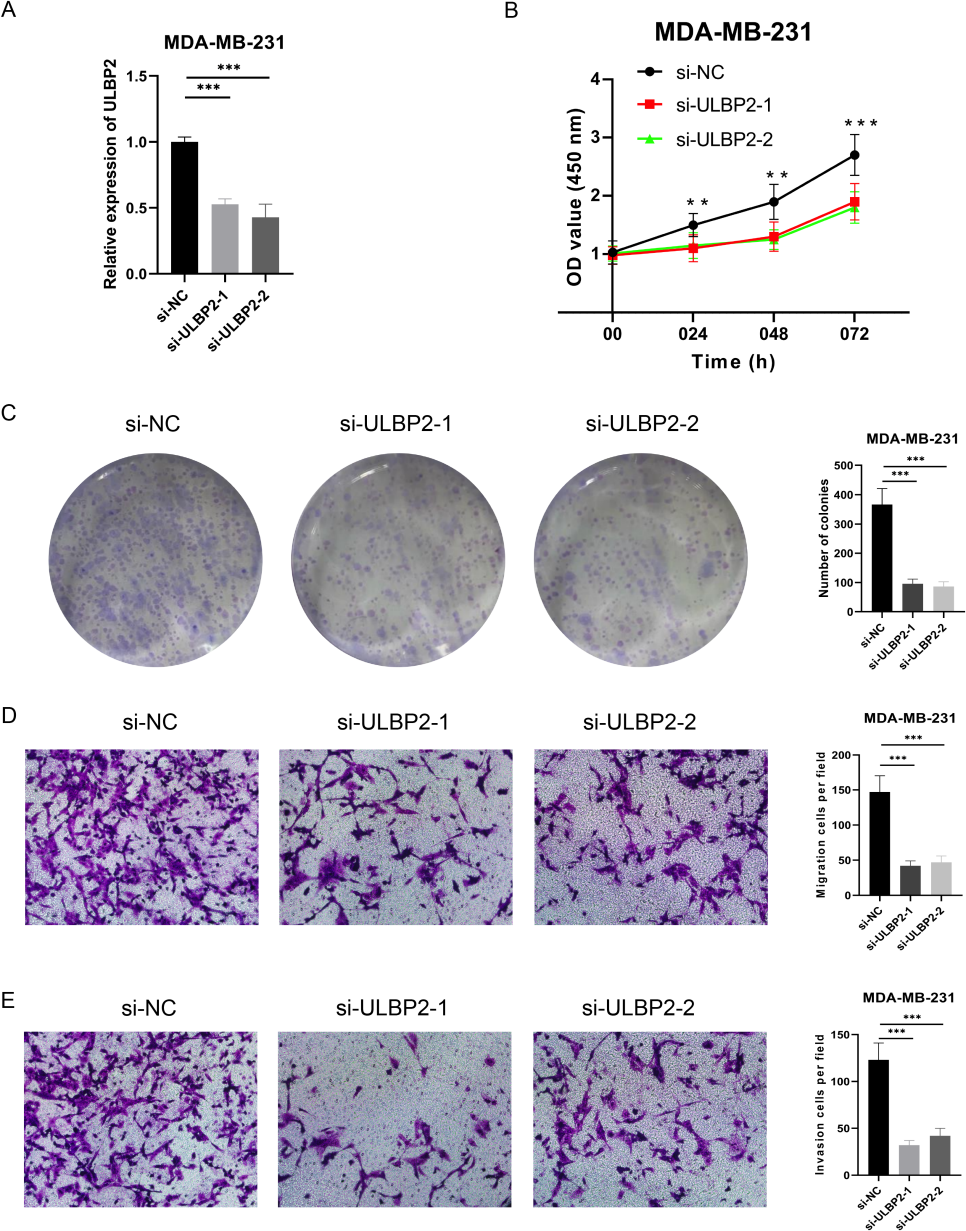
Effect of ULBP2 expression on the proliferation, migration, and invasion of MDA-MB-231 BC cells. **(A)** The knockdown efficiency of ULBP2 by two siRNAs was evaluated using qRT-PCR. **(B)** Cell proliferation was assessed using the CCK-8 assay. **(C)** Colony formation assay was conducted to assess the proliferation/cloning ability. **(D, E)** Transwell migration **(D)** and invasion **(E)** assays were performed to evaluate the invasion and migration capabilities of the cells, respectively. **P < 0.01, ***P < 0.001.

In our previous analysis, we observed that ULBP2 expression is significantly elevated in BC tissues compared to normal tissues, and its high expression is associated with poor prognosis in BC patients. Furthermore, ULBP2 expression was markedly increased in the MDA-MB-231 cell line. Consequently, we selected the MDA-MB-231 cells for knockdown experiments, with the results validated using qRT-PCR. Both siRNAs effectively reduced ULBP2 expression (both p<0.001) (**Figure 9A**). CCK-8 assays demonstrated that knockdown of ULBP2 significantly impaired the proliferative capacity of cancer cells (all p<0.01) (**Figure 9B**). Clonogenic assays further revealed a substantial reduction in the proliferation and clonogenic potential of MDA-MB-231 cells following ULBP2 knockdown (all p<0.001) (**Figure 9C**). Transwell migration and invasion assays provided additional evidence that ULBP2 knockdown significantly decreased the number of migrating and invading cells (all p<0.001) (**Figures 9D, E**). Collectively, our findings demonstrate that silencing ULBP2 suppresses the proliferation, migration, and invasion of BC cells.

### 3.16 Comparison with prior studies

To further validate the performance and robustness of our proposed NRG-based ML models, we conducted a comparative analysis with previously published ML approaches for BC diagnosis and prognosis. As summarized in **Table 3**, these prior studies have employed a variety of biological sources and multimodal imaging modalities to build predictive models using diverse ML algorithms (25–34). For diagnostic modeling, our RF-based model achieved an exceptionally high predictive performance, with an accuracy of 0.999 and an AUC of 1.000. These results significantly outperform previously reported models, including those based on LR (e.g., Zhao et al. with Accuracy = 0.907), XGBoost (e.g., Saadh et al. with AUC = 0.920), and SVM (e.g., Hamyoon et al. with AUC = 0.885). Notably, even models utilizing imaging technologies such as microwave and multiparametric MRI yielded relatively lower AUC values, emphasizing the predictive strength of transcriptome-based NRG features. For prognostic modeling, our model constructed using LASSO and Cox regression demonstrated competitive and consistent performance across different survival time points (1-year AUC = 0.773; 3-year AUC = 0.724; 5-year AUC = 0.683). When compared with other gene signature-based prognostic models—such as RNA modification-related models (e.g., Wang et al., 1-year AUC = 0.694), mitochondrial and lysosome-associated models (e.g., Chen et al., 1-year AUC = 0.738), and redox-associated models (e.g., Wang et al., 1-year AUC = 0.730)—our approach shows comparable or improved predictive capacity. It also maintains performance advantage over vascular mimicry-related models and tertiary lymphoid structure-based predictors, especially in the 3- and 5-year AUC metrics. Taken together, this comparative evaluation demonstrates that our NRG-based models offer competitive or superior diagnostic and prognostic efficacy compared to a broad spectrum of existing ML models. The strong performance, particularly in external validation cohorts, underscores the potential of incorporating immune cell-associated signatures—specifically NK-cell related genes—into clinical decision-support tools for precision oncology.

**Table 3:** Comparison of NRGs-based ML diagnostic and prognostic models with previously published studies.

Research	Characteristics	Models	ML algorithms	Performance evaluation parameters	Paper reference
The current study	NRGs	Diagnosis Model	RF	Accuracy=0.999; AUC=1.000	–
Zhao AR, Kouznetsova VL, Kesari S, et al.	PIWI-interacting RNAs	Diagnosis Model	LR	Accuracy=0.907	(25)
Saadh MJ, Ahmed HH, Kareem RA, et al.	Transcriptomic profiling	Diagnosis Model	XGBoost	Accuracy=0.910; AUC=0.920	(26)
Hamyoon H, Yee Chan W, Mohammadi A, et al.	Ultrasound images	Diagnosis Model	SVM	Accuracy=0.860; AUC = 0.885	(27)
Hu Q, Whitney HM, Giger ML	Multiparametric magnetic resonance images	Diagnosis Model	SVM	AUC = 0.870	(28)
Oliveira BL, Godinho D, O’Halloran M, et al.	Microwave Technology	Diagnosis Model	RF	Accuracy=0.870	(29)
The current study	NRGs	Prognostic model	LASSO and Cox	1-year AUC=0.773; 3-year AUC=0.724; 5-year AUC=0.683	–
Wang T, Wang S, Li Z, et al.	RNA modification signature	Prognostic model	CoxBoost and survival-SVM	1-year AUC=0.694; 3-year AUC=0.696; 5-year AUC=0.682	(30)
Chen H, Wang Z, Shi J, et al.	Mitochondrial and lysosome-related model signature	Prognostic model	CoxBoost and survival-SVM	1-year AUC=0.738; 3-year AUC=0.746; 5-year AUC=0.738	(31)
Zhang X, Li L, Shi X, et al.	Tertiary lymphoid structures	Prognostic model	Enet	1-year AUC=0.659; 2-year AUC=0.736; 3-year AUC=0.668	(32)
Wang T, Wang S, Li Z, et al.	Redox signatures	Prognostic model	RSF	1-year AUC=0.730; 3-year AUC=0.715; 5-year AUC=0.683	(33)
X, Li X, Yang B, et al.	Vascular mimicry signatures	Prognostic model	RFS	3-year AUC=0.631; 5-year AUC=0.646; 10-year AUC=0.719	(34)

## 4 Discussion

As research into the role of NK cells in the TME advances, the clinical application of NK cell-related genes in various cancers is gaining increasing attention (14, 35, 36). In this study, we developed and validated a ML diagnostic model based on the RF algorithm, utilizing seven NRGs which were ULBP2, CCL5, PRDX1, IL21, NFATC2, CD2, and VAV3. The model demonstrated high accuracy across different datasets and clinical subgroups. Furthermore, using these seven NRGs, we constructed a prognostic ML model that exhibited strong predictive capability, effectively forecasting the survival outcomes of BC patients. Our findings highlight the crucial role of NRGs in BC diagnosis and prognosis, shedding light on their potential utility in precision medicine. Previously, Zundong et al. constructed a prognostic risk model using five NRGs in triple-negative breast cancer (TNBC) patients (37). In comparison to the study conducted by Zundong et al., our work introduces a novel integration of NRGs and ML methods to develop a diagnostic model for BC. This innovative approach not only enhances the early screening and diagnosis of BC but also contributes to a deeper understanding of the role of NRGs in the pathogenesis of BC. In terms of predicting survival outcomes, our prognostic model includes a larger sample size and places greater emphasis on the correlation between risk scores and clinical indicators in BC patients. These improvements make our model more robust and enhance its potential for broader clinical application. Currently, Oncotype DX Breast Recurrence Score plays a significant role in predicting the recurrence risk and chemotherapy benefits for BC patients, and its widespread application has also driven a shift in treatment paradigms (38–40). However, Oncotype DX is primarily designed for early-stage BC patients who are hormone receptor-positive, HER2-negative, and lymph node-negative, limiting its applicability in other subtypes and stages of BC. In contrast, our prognostic model explores clinical applications across various stages and types of BC, addressing this limitation. Moreover, while Oncotype DX mainly focuses on gene expression traits related to tumor proliferation and invasion, our model centers on NK cell-related features. This may offer a greater clinical advantage in predicting responses to immunotherapy, particularly in BC subtypes such as TNBC, which show higher sensitivity to immune treatments. Therefore, integrating NK cell characteristics into clinical decision-making could complement existing tools like Oncotype DX or provide an alternative when traditional methods are less effective.

ULBP2 (UL16-binding protein 2) is one of the ligands for the natural killer group 2 member D (NKG2D) receptor, and its expression is up-regulated in various stress, oncogenic, or infected cells, where it binds to NKG2D, thereby inducing cytotoxicity and cytokine production by NK cells (41). Interestingly, our current study reveals that ULBP2 is not only highly expressed in BC patients but also correlates with poorer prognosis. Furthermore, we validated through functional assays that its elevated expression promotes the proliferation, migration, and invasion of BC cells. Studies have reported that soluble ULBP2, as a ligand of NKG2D, suppresses the expression of NKG2D and inhibits NK cell activity, thereby allowing tumor cells to escape immune surveillance and promoting immune evasion (42). CCL5 (C-C motif chemokine ligand 5) is a chemokine that primarily acts on immune cells. By binding to the CCR5 receptor, it contributes to an increased risk of BC recurrence by facilitating the recruitment of tumor-associated macrophages (43). Furthermore, elevated expression of CCL5 is associated with poor prognosis in BC, particularly in its role in promoting tumor invasiveness and metastasis (44). Interestingly, our current study found that patients with high CCL5 expression exhibited better prognoses. This suggests that the role of CCL5 in the TME is multifaceted. In addition to its well-documented involvement in promoting tumor cell migration and invasion, CCL5 may also enhance anti-tumor immune responses by modulating immune activity and promoting immune cell infiltration. These findings indicate that CCL5 could have a dual role in both immune evasion and immune surveillance. Further investigation is warranted to elucidate the specific mechanisms of CCL5 across different BC subtypes. The role of PRDX1 (peroxiredoxin 1) in BC has garnered widespread attention. In BC cells, PRDX1 may prevent oxidative stress-induced loss of ERα through its antioxidant function, potentially contributing to the maintenance of the ER-positive phenotype in BC (45). The expression level of PRDX1 not only affects cell growth and survival but is also associated with the invasiveness and metastatic potential of BC. Studies have shown that down-regulation of PRDX1 significantly inhibits the growth rate of BC cells, and *in vivo*, PRDX1-deficient MCF-7 cells exhibit delayed tumor growth upon transplantation (46). IL21 (Interleukin-21) is a cytokine that can influence the development and progression of BC through various mechanisms that regulate the immune system. The expression of IL21 is closely associated with processes such as the proliferation, migration, and immune evasion of BC cells (47). NFATC2 (nuclear factor of activated T cells 2) is a transcription factor that plays a critical role in the activation of immune cells. Research indicates that NFATC2 regulates the expression of matrix metalloproteinase 13 (MMP13) in BC cells through interactions with other proteins, thereby promoting the invasiveness of cancer cells (48), which provides a new therapeutic target for BC treatment. CD2 is an important cell adhesion molecule primarily expressed on T cells and NK cells, playing a crucial role in the formation and organization of the immunological synapse. Studies have shown that CD2 overexpression can inhibit the activation of nitrogen metabolism pathways and suppress M2 polarization of macrophages, thereby preventing brain metastasis of BC (49). Additionally, the interaction between CD2 and CD58 is vital in the early stages of immune responses, as modulating this interaction can influence the intensity and nature of immune reactions (50). By regulating CD2-associated signaling pathways, the immune system’s ability to recognize and eliminate tumor cells can be enhanced, offering new perspectives and potential strategies for BC treatment (51). VAV3 (Vav guanine nucleotide exchange factor 3) is a member of the Rho GTPase guanine nucleotide exchange factor family and plays a pivotal role in cytoskeletal remodeling, cell motility, and oncogenic signal transduction. Its overexpression in BC has been reported to drive tumor cell proliferation, invasion, and metastasis via the Rac1/MAPK signaling pathway (52). Moreover, studies have indicated that VAV3 expression correlates significantly with poor prognosis, making it not only a diagnostic marker but also a prognostic indicator (53). Among the NRGs identified by the SHAP interpretability analysis, VAV3 consistently exhibited a high contribution to the RF diagnostic model in both the TCGA and GEO cohorts, highlighting its potential as a key biomarker for BC detection. Clinically, the high SHAP value of VAV3 underscores its importance in the machine learning model and suggests that VAV3 could serve as a molecular marker for early identification of aggressive subtypes of BC, particularly those with high metastatic potential. From a therapeutic perspective, targeting the VAV3-mediated signaling pathway may offer a novel strategy for tailored treatment in high-VAV3-expressing patients. Additionally, as VAV3 plays a role in immune signaling modulation within the tumor microenvironment, its expression may also influence response to immunotherapies, a hypothesis warranting further investigation. Overall, these NRGs not only play a pivotal role in the immune evasion mechanisms of BC but are also closely associated with patient survival prognosis, providing a foundation for the development of ML-based diagnostic and prognostic models.

To explore the potential factors influencing the prognostic differences between high-risk and low-risk groups, we identified DEGs and performed functional enrichment analysis between the two risk groups. The results suggest that the high-risk group may experience more complex immune responses and changes in the cellular environment, potentially rendering it more susceptible to infections or exhibiting abnormal immune activation, thereby increasing the risk of immune evasion or inflammation-related diseases. In contrast, the low-risk group may rely on stable immune surveillance mechanisms, demonstrating a stronger immune response capability, which could contribute to better tumor suppression and prognosis. In the TME of BC, the activity of NK cells is regulated by various factors, such as TGF-β, soluble HLA-G, prostaglandin E_2_, adenosine, extracellular vesicles, and miRNAs (54). These factors can both inhibit the anti-tumor activity of NK cells and induce their pro-angiogenic polarization, thereby supporting tumor progression. The interactions between NK cells and other immune cells are also crucial. Studies have shown that the interplay between NK cells, T cells, myeloid-derived suppressor cells, and tumor-associated macrophages can significantly influence the dissemination, immune editing, and therapeutic outcomes of BC (55). Therefore, it is essential to explore the differences in the TME between high-risk and low-risk groups. In terms of cancer hallmark features, tumor cells in the high-risk group are enriched for pathways related to the G2M checkpoint, tumor proliferation characteristics, DNA replication, MYC target genes, and cellular responses to hypoxia, all of which are significantly positively correlated with risk scores. This suggests that tumor cells in the high-risk group possess enhanced proliferative capacity, genomic instability, and adaptability, enabling them to sustain growth even in adverse environments. These features are typically associated with increased tumor invasiveness, metastatic potential, and resistance to therapy (56, 57). Furthermore, the high-risk group shows significant enrichment in the Oxeiptosis pathway, indicating a distinctive regulation of oxidative stress-related death signals. In contrast, the low-risk group is primarily enriched in pathways related to necroptosis, immunogenic cell death, and pyroptosis, which are typically associated with inflammation and immune activation (58–60). Immunological analyses reveal a marked reduction in the infiltration levels of CD8+ T cells, NK cells, tumor-infiltrating lymphocytes, CD4+ T cells, and follicular helper T cells in the high-risk group, accompanied by a general downregulation of immune functions. Moreover, the expression of MHC molecules, chemokines and receptors, and ICGs is suppressed. Taken together, these findings demonstrate that the high-risk group exhibits enhanced tumor proliferative capabilities, immune evasion mechanisms, and weakened immune surveillance, which contribute to its increased ability to escape immune system clearance, leading to poorer clinical outcomes. Future studies could further explore how targeting the regulation of cell death pathways and restoring anti-tumor immune responses can improve treatment outcomes for high-risk patients. Additionally, intervention strategies targeting key pathways, including MYC signaling, hypoxic adaptation, and DNA damage repair, may emerge as critical directions for personalized therapy in the future.

In recent years, significant progress has been made in the field of immunotherapy for BC. As an emerging treatment modality, immunotherapy has been approved as a first-line treatment for metastatic TNBC with PD-L1 overexpression. However, the clinical activity of immune checkpoint inhibitors as a monotherapy in advanced BC has been somewhat limited. Consequently, increasing attention is being paid to combination therapies, particularly in the rapidly evolving early-stage disease setting (61). The IMpassion130 phase III trial compared chemotherapy combined with atezolizumab to chemotherapy plus placebo, revealing positive overall survival outcomes in PD-L1-positive TNBC patients. This underscores the need to further expand the patient population that may benefit from immunotherapy, highlighting the importance of discovering and implementing new biomarkers in this context (62). Additionally, advances in BC immunotherapy are also reflected in the deeper exploration of the tumor immune microenvironment. For HER2-negative patients carrying BRCA1 or BRCA2 mutations, PARP inhibitors have been associated with improved overall survival in certain subgroups (63). Therefore, the progress of BC immunotherapy is not only reflected in the development of new drugs and new therapies, but also in the in-depth study of patient selection and biomarkers, which provide new directions and possibilities for future treatment strategies. Our study assessed the potential response of BC patients to immunotherapy through IPS and the evaluation of immune evasion mechanisms. The results revealed that, compared to the low-risk group, the IPS scores in the high-risk group was significantly lower, and this trend persisted despite differences in the expression of PD-1 and CTLA-4. Moreover, although there was no significant difference in the TIDE score between the two groups, the high-risk group exhibited lower expression of genes such as IFNG, Merck18, CD274, and CD8. Additionally, the Dysfunction score was lower and the Exclusion score was higher in the high-risk group. These characteristics suggest that high-risk breast cancer patients may possess a stronger immune evasion capability, leading to a poorer response to ICIs therapy. Further analysis of data from the TCGA cohort and the IMvigor210 real-world study cohort revealed that, although statistical significance was not reached in the TCGA cohort, the low-risk group demonstrated a higher response rate to immunotherapy. This finding emphasizes the close association between the TME status and immunotherapy efficacy, suggesting that high-risk breast cancer patients may exhibit limited responses to ICIs due to a more suppressive immune microenvironment. Furthermore, in the GSE4779 and GSE25066 cohorts, the proportion of BC patients who achieved pCR after neoadjuvant chemotherapy was relatively low. This phenomenon suggests that higher risk scores are associated with stronger chemotherapy resistance. Although patients in the high-risk group may derive lower benefits from immunotherapy and chemotherapy, a drug sensitivity analysis of 235 drugs revealed several potential therapeutic agents that could benefit high-risk patients. Five drugs that were significantly negatively correlated with risk scores include Thapsigargin, Docetaxel, AKT Inhibitor VIII, Pyrimethamine, and Epothilone B, which may hold greater therapeutic potential for high-risk patients. Thapsigargin, in particular, shows promise in BC treatment, especially due to its unique calcium signaling mechanism that induces apoptosis in tumor cells. However, toxicity and targeting remain critical challenges in current research. In the future, combining prodrug design, nanodelivery systems, and combination therapy strategies may position Thapsigargin or its derivatives as a new therapeutic option for BC treatment (64). Docetaxel has demonstrated excellent efficacy and tolerability in the treatment of BC across different stages and subtypes, making it a crucial component of breast cancer chemotherapy. When combined with cyclophosphamide and trastuzumab for neoadjuvant therapy in HER2-positive BC, docetaxel has shown a high pCR rate, suggesting that this combination regimen could be an effective option for preoperative treatment of HER2-positive BC (65). Additionally, the sequential use of docetaxel with doxorubicin and cyclophosphamide in early-stage BC has also proven to be feasible for neoadjuvant therapy. Studies have reported a clinical response rate as high as 90%, with the majority of patients being able to undergo breast-conserving surgery, highlighting the potential of this regimen in early-stage BC treatment (66). In metastatic BC, the combination of docetaxel and gemcitabine as first-line treatment has shown promising efficacy and tolerability (67). Overall, our study uncovers the immune evasion mechanisms in high-risk patients and their impact on treatment response. Through drug screening, we have identified potential novel therapies, offering new directions and strategies for the future of personalized BC treatment.

This study developed a ML-based diagnostic and prognostic model utilizing NK cell-related genes, providing a novel approach for personalized medicine in BC. However, there are several limitations. First, the data primarily came from the TCGA and GEO databases, which may introduce ethnic and regional biases, necessitating validation with broader population data. Second, the inclusion of clinical variables remains limited, as factors such as treatment regimens and lifestyle were not considered. Furthermore, the complexity of the machine learning model may reduce its clinical interpretability, and future studies could integrate methods such as SHAP to enhance model transparency. Future research can be advanced in several key directions. Firstly, integrating multi-omics data to enhance the accuracy and generalizability of the model. Secondly, incorporating longitudinal data to better predict the progression and recurrence of BC. Thirdly, investigating the role of NK cell-related genes in immunotherapy to refine and optimize personalized treatment strategies. Furthermore, validating the clinical applicability of the model through clinical trials is crucial to facilitate its integration into real-world medical decision-making. In terms of clinical implementation, the proposed ML-based diagnostic and prognostic models can be embedded into hospital electronic medical systems as decision-support tools. Specifically, the RF diagnostic model can assist clinicians in the early identification of BC by analyzing gene expression profiles derived from biopsy or blood samples, which could be particularly beneficial for patients at early stages or with ambiguous imaging findings. The prognostic risk score model allows stratification of patients into different risk groups, helping guide treatment intensity—especially in selecting candidates for chemotherapy or immunotherapy. The integration of a nomogram that combines clinical factors with model-derived risk scores enhances interpretability and usability in clinical practice. While retrospective validation shows strong potential, future prospective studies and integration with electronic health record systems will be essential for full clinical translation. Despite the limitations of the current study, its findings lay a critical theoretical foundation for future research on the immune mechanisms of BC and the advancement of personalized medicine.

## 5 Conclusion

In this study, we developed and validated ML-based diagnostic and prognostic models for BC using NRGs. The diagnostic model, built using the RF algorithm, demonstrated high accuracy across multiple datasets, offering a reliable tool for BC detection. The prognostic model effectively stratified patients into high-risk and low-risk groups, highlighting differences in survival outcomes, immune characteristics, and treatment responses. High-risk patients exhibited enhanced tumor proliferation, immune evasion, and reduced immune cell infiltration, which correlated with poorer clinical outcomes. Moreover, the high-risk group showed lower IPS values and a weaker response to immune checkpoint inhibitors, underscoring the importance of precise risk stratification in treatment planning. These findings reveal the critical role of NRGs in BC progression and underscore the potential of integrating ML-based NRG models into precision oncology to improve diagnostic accuracy, guide personalized treatment, and ultimately enhance patient outcomes. Further clinical validation and prospective studies are warranted to fully realize their translational potential.

## Data Availability

The raw data supporting the conclusions of this article will be made available by the authors, without undue reservation.

## Glossary

BC: breast cancer
TME: tumor microenvironment
NK: natural killer
CAR-NK: chimeric antigen receptor NK
CARs: chimeric antigen receptors
ML: machine learning
TCGA: The Cancer Genome Atlas
GEO: Gene Expression Omnibus
DEGs: differentially expressed genes
OS: overall survival
LR: logistic regression
XGBoost: extreme gradient boosting
LightGBM: light gradient boosting machine
RF: random forest
AdaBoost: adaptive boosting
DT: decision tree
GB: gradient boosting
GNB: gaussian naive bayes
CNB: complement naive bayes
MLP: multi-layer perceptron neural networks
SVM: support vector machine, KNN, k-nearest neighbors
AUC: area under the curve
PPV: positive predictive value
NPV: negative predictive value
DCA: Decision Curve Analysis
SHAP: SHapley Additive exPlanations
PCA: principal component analysis
t-SNE: t-distributed stochastic neighbor embedding
KM: Kaplan-Meier
ROC: receiver operating characteristic
GO: Gene Ontology
KEGG: Kyoto Encyclopedia of Genes and Genomes
GSEA: Gene Set Enrichment Analysis
DO: Disease Ontology
ssGSEA: single sample gene set enrichment
MHC: major histocompatibility complex
ICGs: immune checkpoint genes
IPS: Immunophenoscore
TCIA: The Cancer Immunome Atlas
TIDE: Tumor Immune Dysfunction and Exclusion
GDSC: Drug Sensitivity in Cancer
IC50: 50% inhibitory concentration
TMB: tumor mutational burden
MSI: microsatellite instability
ITH: intratumor heterogeneity
TISCH: Tumor Immune Single-cell Hub
qRT-PCR: quantitative real-time PCR
OD: optical density
ER: estrogen receptor
PR: progesterone receptor
HER2: human epidermal growth factor receptor 2
pCR: pathological complete response
SNV: single nucleotide variations
TNBC: triple-negative breast cancer
ULBP2: UL16-binding protein 2
NKG2D: natural killer group 2 member D
CCL5: C-C motif chemokine ligand 5
PRDX1: peroxiredoxin 1
IL21: Interleukin-21
NFATC2: nuclear factor of activated T cells 2
MMP13: matrix metalloproteinase 13
CD2: cluster of differentiation 2
VAV3: Vav guanine nucleotide exchange factor 3.
