# Supplementary figures and images for: Machine learning-based diagnostic and prognostic models for breast cancer: a new frontier on the clinical application of natural killer cell-related gene signatures in precision medicine
Source: Front Immunol. 2025 May 27;16:1581982. doi: 10.3389/fimmu.2025.1581982 (PMC12149121; doi:10.3389/fimmu.2025.1581982)

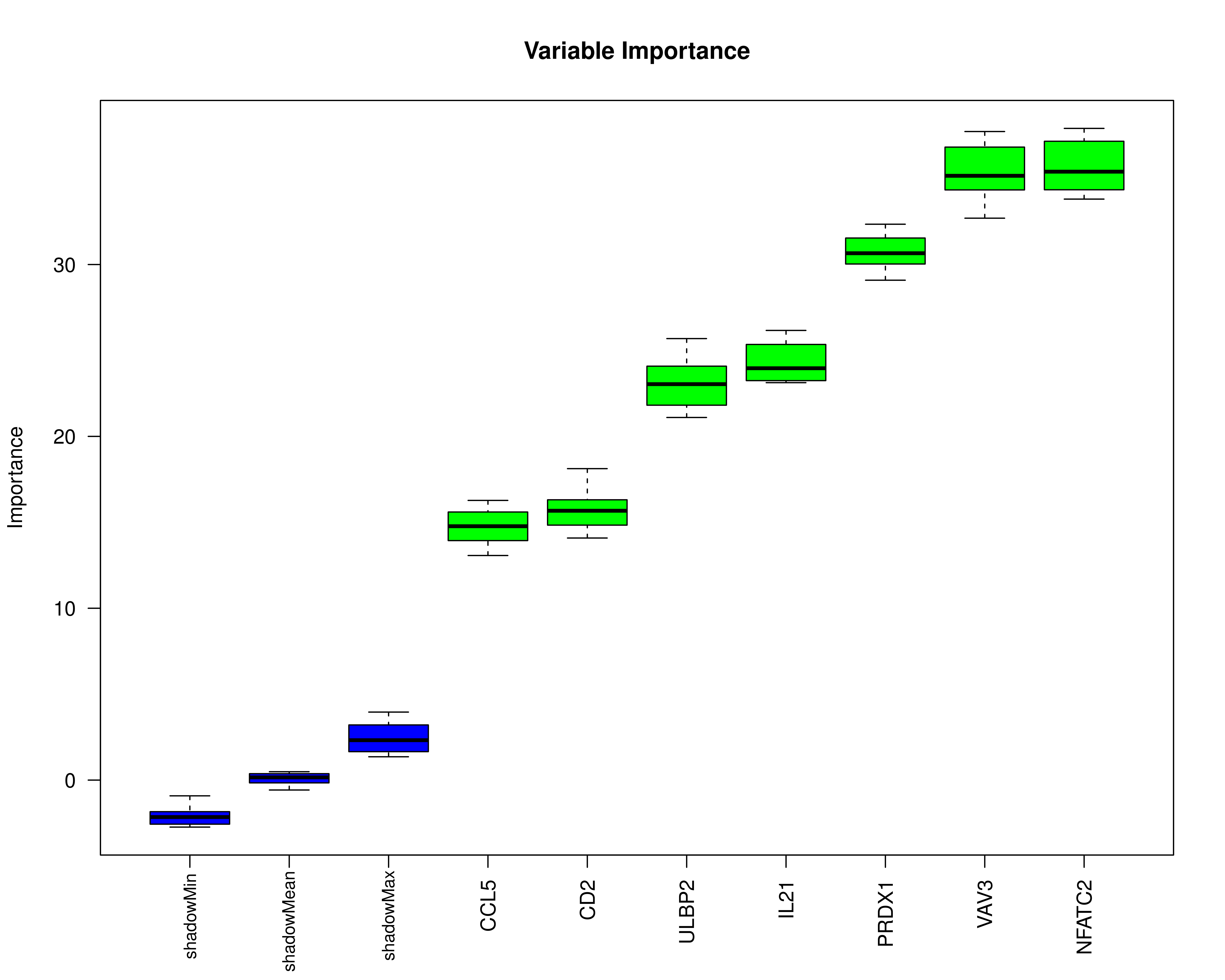

Supplement: Supplementary file 1 [file Image1.tif]

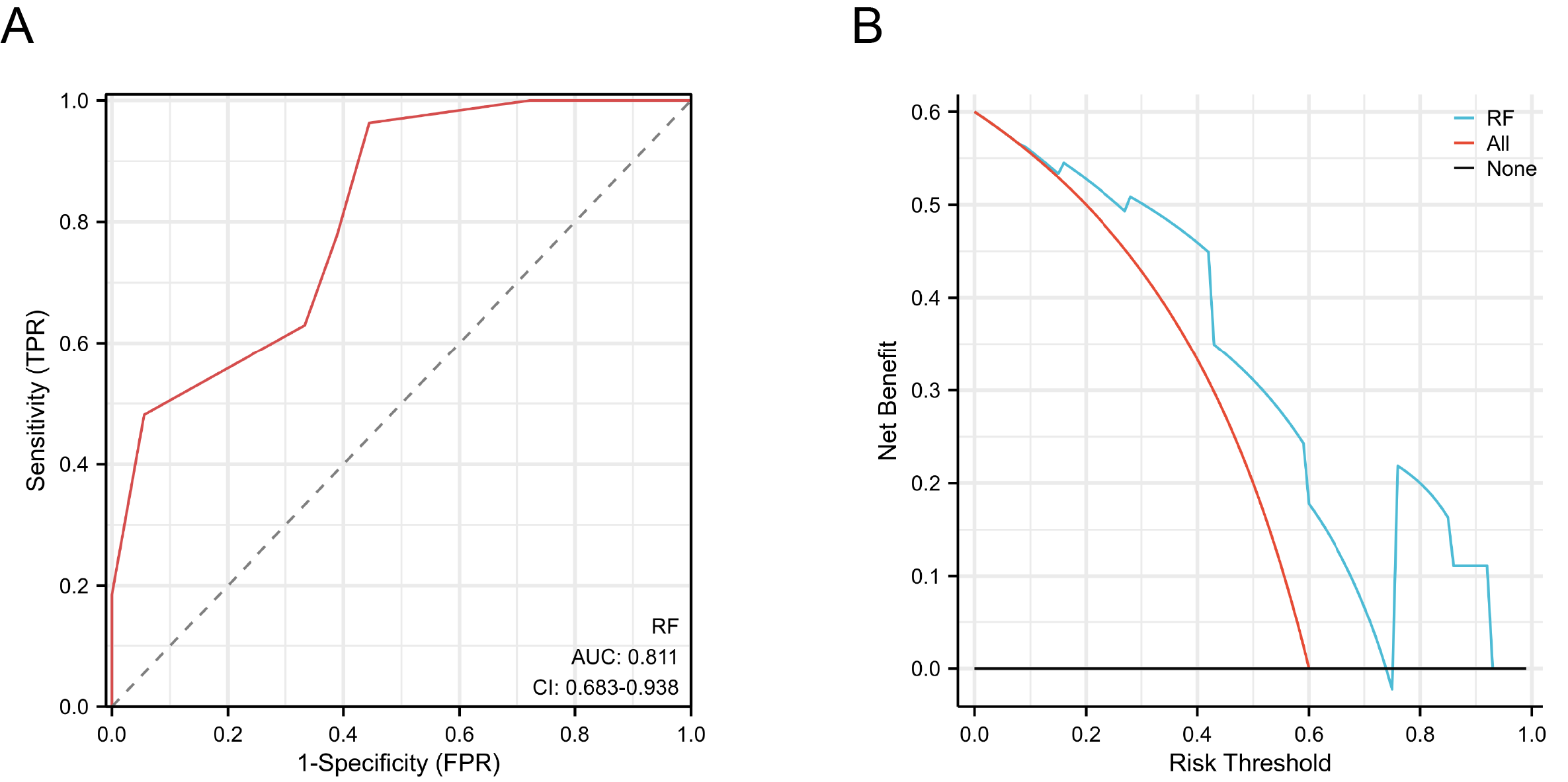

Supplement: Supplementary file 2 [file Image2.tif]

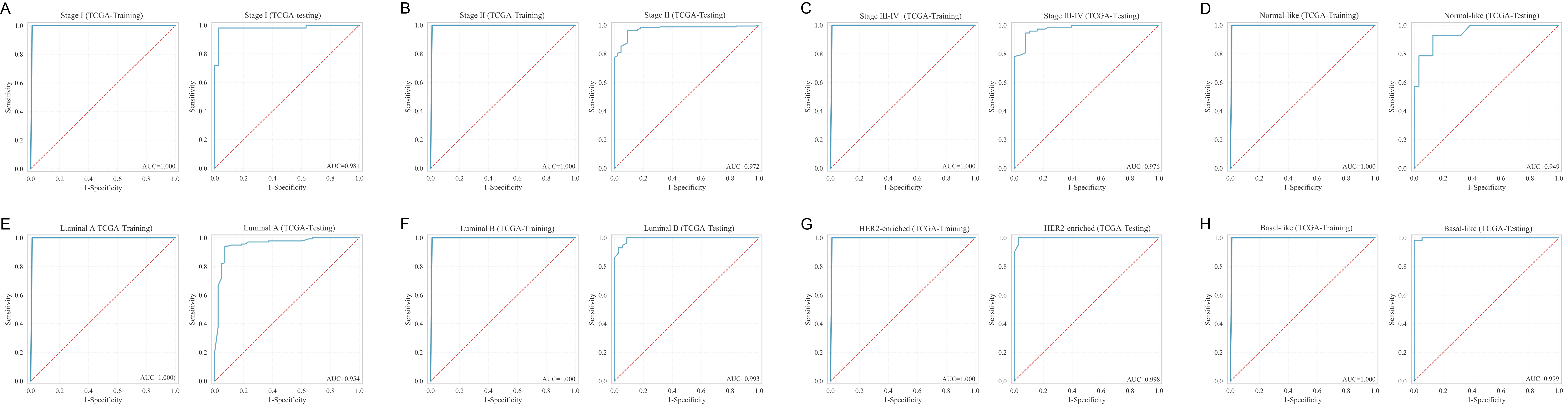

Supplement: Supplementary file 3 [file Image3.tif]

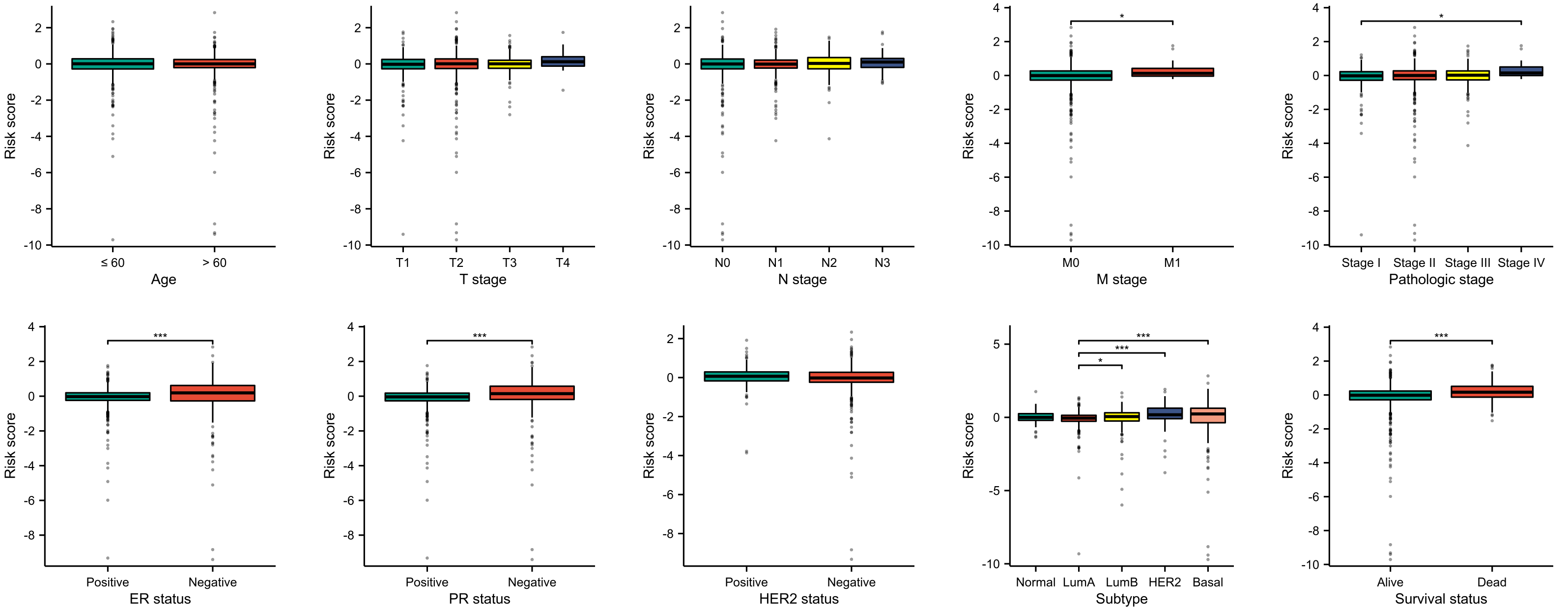

Supplement: Supplementary file 4 [file Image4.tif]

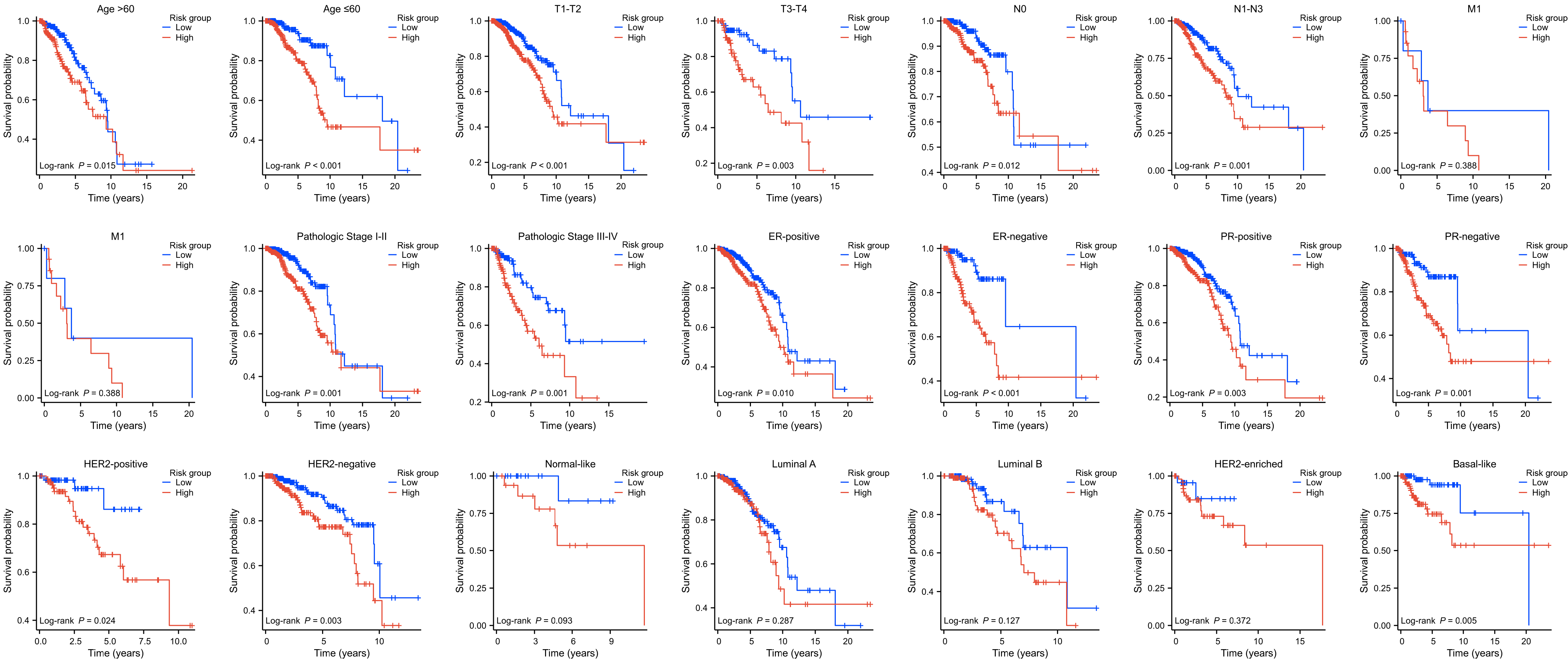

Supplement: Supplementary file 5 [file Image5.tif]

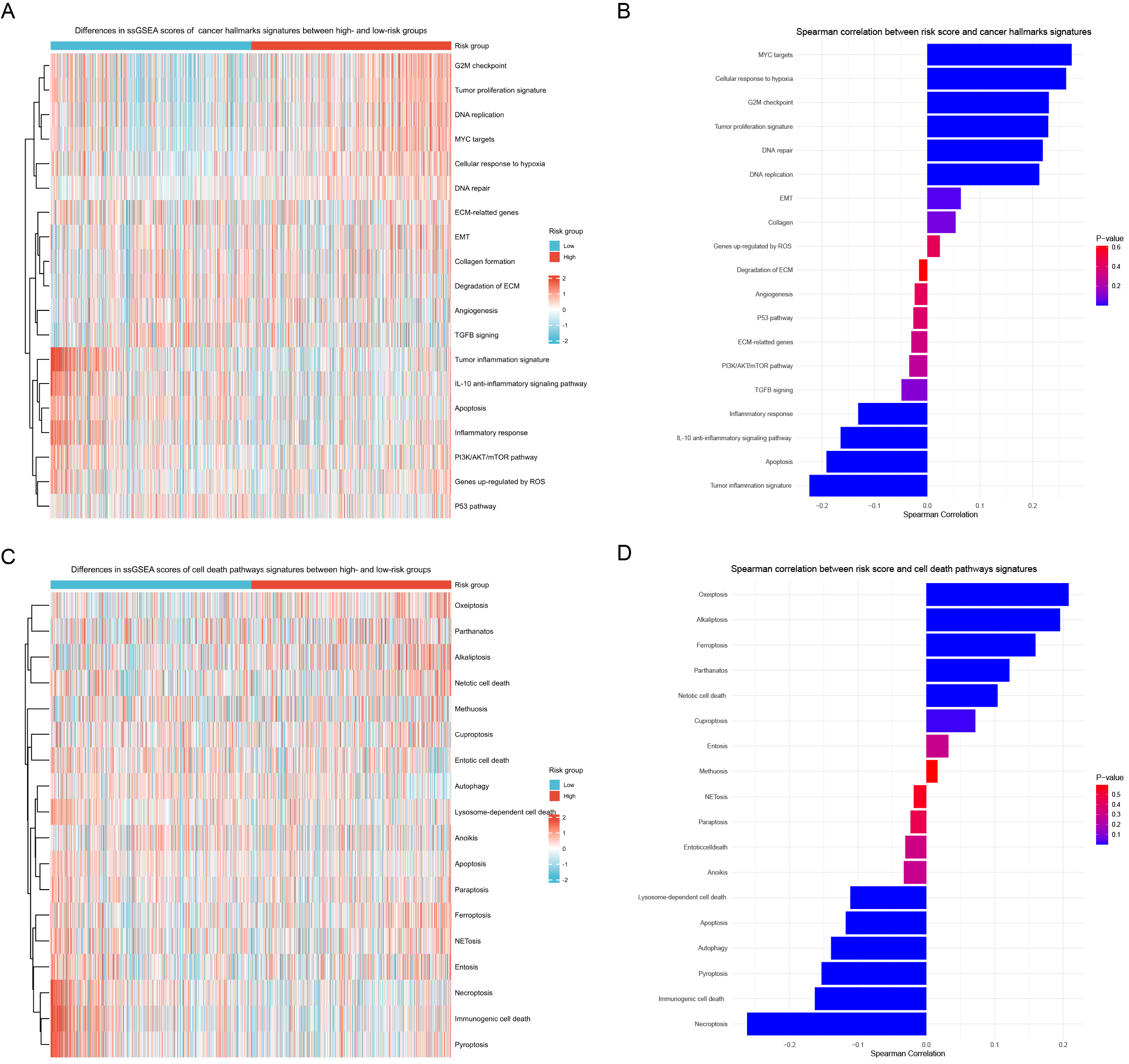

Supplement: Supplementary file 6 [file Image6.tif]

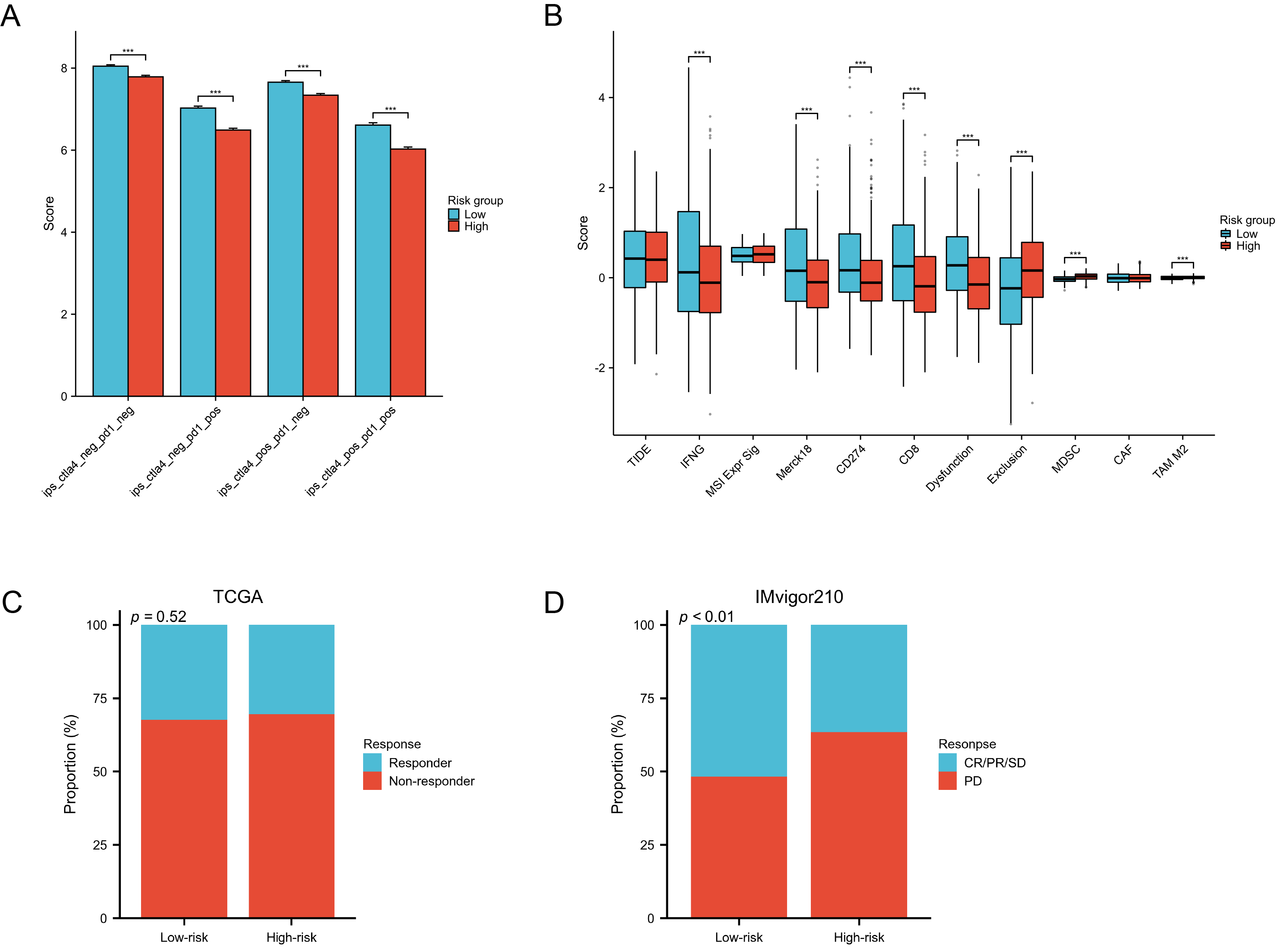

Supplement: Supplementary file 7 [file Image7.tif]

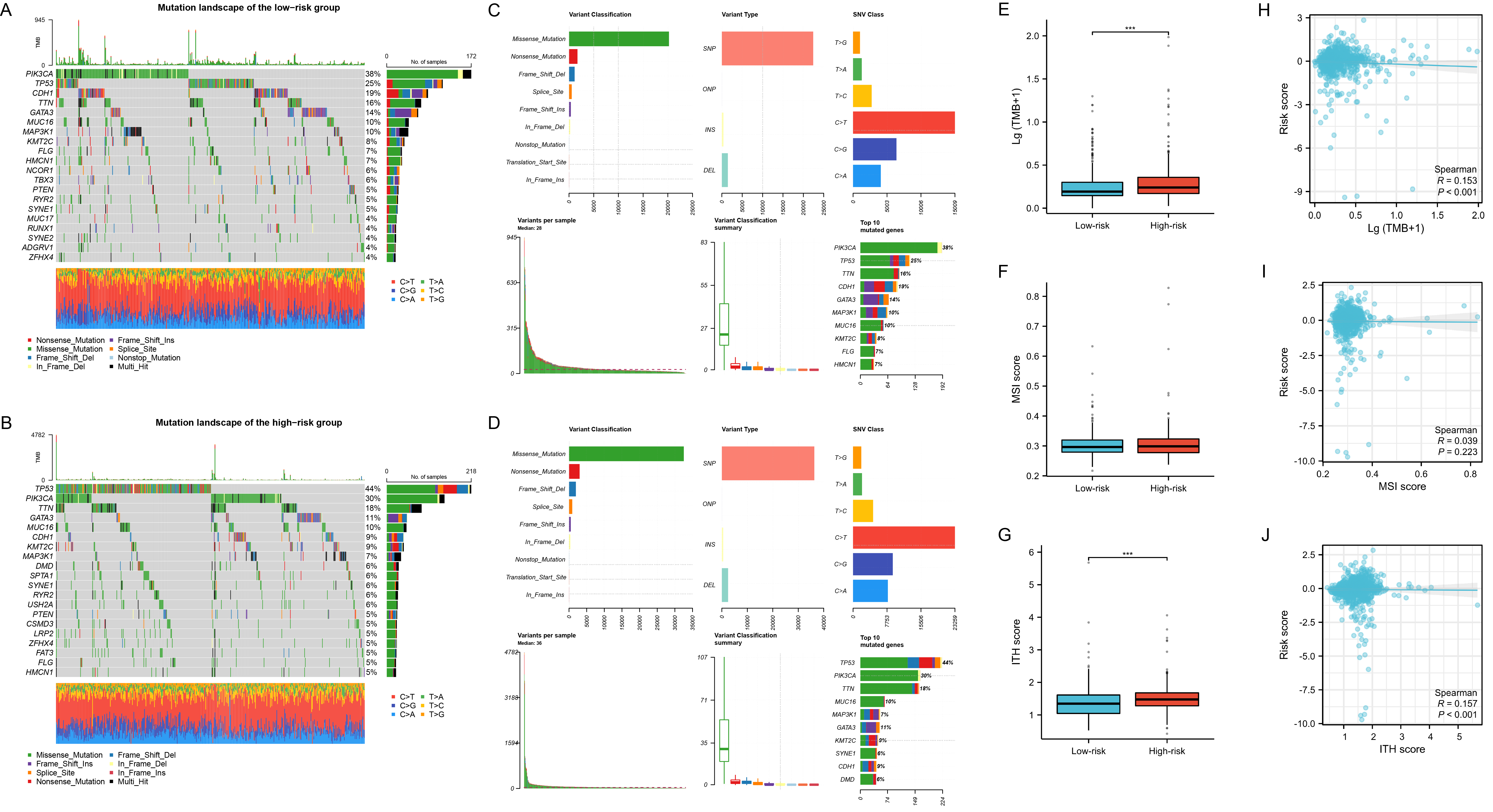

Supplement: Supplementary file 8 [file Image8.tif]

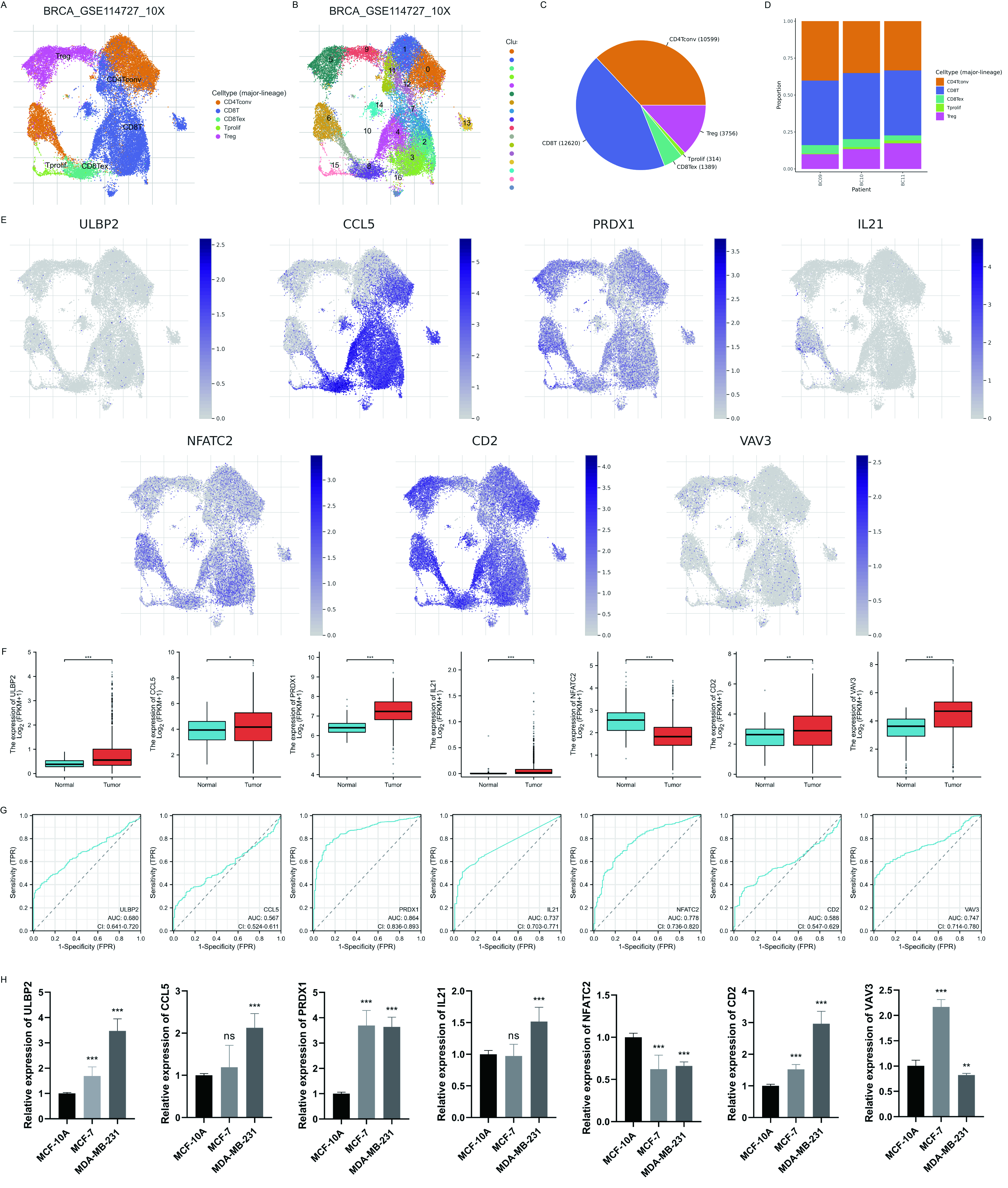

Supplement: Supplementary file 9 [file Image9.tif]

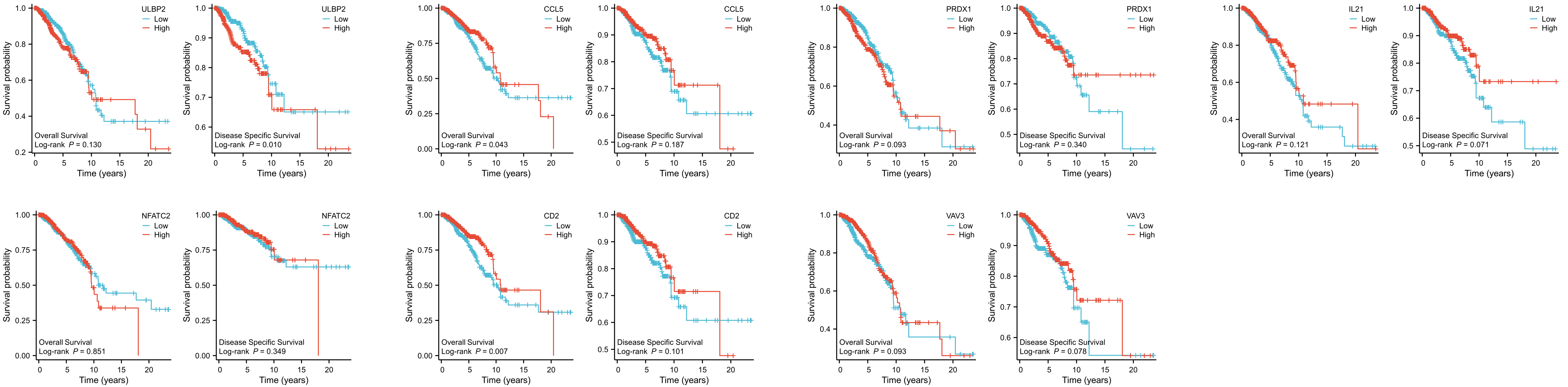

Supplement: Supplementary file 10 [file Image10.tif]
