# Supplementary material for: Machine learning-based diagnostic and prognostic models for breast cancer: a new frontier on the clinical application of natural killer cell-related gene signatures in precision medicine
Source: Front Immunol. 2025 May 27;16:1581982. doi: 10.3389/fimmu.2025.1581982 (PMC12149121; doi:10.3389/fimmu.2025.1581982)
Supplement: Supplementary file 11 [file Table1.docx]

**Supplementary Figure S1.** The importance of the features of the diagnostic models determined by the Boruta algorithm.

**Supplementary Figure S2.** ROC analysis (A) and DCA (B) of the RF diagnostic model for a clinical validation cohort from patients.

**Supplementary Figure S3.** Clinical application of the RF diagnostic model in different pathologic stages and PAM50 subtypes of BC.

**Supplementary Figure S4.** Differences in risk scores among different clinical subgroups of BC. *P < 0.05, ***P < 0.001.

**Supplementary Figure S5.** KM survival analysis of the OS between the two risk groups in various clinical subgroups.

**Supplementary Figure S6.** Differences in cancer hallmarks and cell death pathways in the two risk groups. **(A)** Heatmap shows the enrichment of cancer hallmarks in the two risk groups. **(B)** Spearman correlation analysis between the risk scores and enrichment scores of the cancer hallmarks. **(C)** Heatmap shows the enrichment of cell death pathways in the two risk groups. **(D)** Spearman correlation analysis between the risk scores and enrichment scores of the cell death pathways.

**Supplementary Figure S7.** Immunotherapy response prediction. **(A)** Differences in IPS scores between the two risk groups. **(B)** TIDE analysis between the two risk groups. **(C, D)** Proportion of patients who non-respond and respond to immunotherapy in TCGA (C) and IMivgor210 (D) cohorts.

**Supplementary Figure S8.** Mutation analysis between the two risk groups. **(A, B)** Waterfall plots of somatic mutation features established with low- (A) and high-risk (B) groups. **(C, D)** Mutation status statistics in low- (C) and high-risk (D) groups. **(E-G)** Differences in TMB (E), MSI (F), and ITH (G) scores between the two risk groups. **(H-J)** Spearman correlation analysis between the risk scores and TMB (H), MSI (I), and ITH (J) scores. ***P < 0.001.

**Supplementary Figure S9.** Single cell and differential expression analyses of the 7NRGs. (**A**) Annotation of the cell types. (**B**) Annotation of the clusters. (**C, D**) Quantities and proportions of different cell types in the GSE114727_10X dataset. (**E**) Percentages and expressions of the 7 NRGs. (**F**) Differential expression of the 7NRGs between BC and normal tissues. (**G**) Diagnostic values of the 7 NRGs in BC. (**H**) Validation of the differential expression of the 7 NRGs in cell lines using qRT-PCR. NS indicates no statistical difference, *P < 0.05, **P < 0.01, ***P < 0.001.

**Supplementary Figure S10.** KM survival analysis for the OS and DSS between high- and low-expression groups of the seven NRGs for models construction.
